# Enhanced secretion of the amyotrophic lateral sclerosis ALS-associated misfolded TDP-43 mediated by the ER-ubiquitin specific peptidase USP19

**DOI:** 10.1007/s00018-025-05589-w

**Published:** 2025-02-13

**Authors:** Flavien Picard, Takashi Nonaka, Edwige Belotti, Alexis Osseni, Elisabeth Errazuriz-Cerda, Coline Jost-Mousseau, Emilien Bernard, Agnès Conjard-Duplany, Delphine Bohl, Masato Hasegawa, Cédric Raoul, Thierry Galli, Laurent Schaeffer, Pascal Leblanc

**Affiliations:** 1 Institut NeuroMyoGène-PGNM, Faculté de Médecine Rockefeller, Université Claude Bernard Lyon, Lyon, France; 2 Department of Brain and Neurosciences, Tokyo Metropolitan Institute of Medical Science, Setagaya-Ku, Tokyo, 156-8506 Japan; 3 Plateforme d’imagerie CIQLE, Lyon, France; 4 Sorbonne Université, Institut du Cerveau—ICM, INSERM, CNRS, AP-HP, Hôpital de La Pitié-Salpêtrière, Paris, France; 5 Lyon ALS Reference Center, Hôpital Neurologique Pierre Wertheimer, Hospices Civils de Lyon, Université de Lyon, 59 Boulevard Pinel, 69677 Bron, France; 6 INM, Univ Montpellier, INSERM, Montpellier, France, 34095 Montpellier, France; 7 ALS reference center, Univ Montpellier, CHU Montpellier, Montpellier, France; 8 Université Paris Cité, Institute of Psychiatry and Neuroscience of Paris, INSERM U1266, Membrane Traffic in Healthy & Diseased Brain, 75014 Paris, France; 9 GHU Paris Psychiatrie & Neurosciences, Paris, France; 10 Groupement Hospitalier Est, Hospices Civils de Lyon, Bron, France

**Keywords:** ALS, TDP-43, Ubiquitin peptidase, USP19, Release/secretion, Aggregates, Endosomes, Autophagy, Autophagosomes, Misfolding

## Abstract

Proteinopathies, such as amyotrophic lateral sclerosis (ALS), are marked by the accumulation of misfolded proteins that disrupt cellular processes. Eukaryotic cells have developed protein quality control systems to eliminate these aberrant proteins, but these systems often fail to differentiate between normal and misfolded proteins. In ALS, pathological inclusions primarily composed of misfolded TDP-43 are a hallmark of the disease. Recently, a novel unconventional secretion process called misfolding-associated protein secretion (MAPS) has been discovered to selectively export misfolded proteins. USP19, an Endoplasmic Reticulum-associated ubiquitin peptidase, plays a crucial role in this process. In this study, we investigated the impact of ER-anchored USP19 on the secretion of misfolded TDP-43. Here we found that USP19 overexpression significantly promotes the secretion of soluble and aggregated misfolded TDP-43, requiring both ER anchoring and ubiquitin peptidase activity. Characterization of the cellular and molecular mechanisms involved in this process highlighted the importance of early autophagosomal and late endosomal/amphisomal compartments, while lysosomes did not play a key role. By using dominant-negative mutants and small interfering RNAs, we identified that USP19-mediated secretion of misfolded TDP-43 is modulated by key factors involved in cellular trafficking and secretion pathways, such as ATG7, the ESCRT-O HGS/HRS, the Rab GTPases RAB11A, RAB8A, and RAB27A, and the v-SNARE VAMP7. We also confirmed the crucial role of the DNAJC5/CSPα cochaperone. Overall, this study provides new insights into how cells manage the secretion of misfolded TDP-43 proteins and potentially opens new avenues for therapeutic interventions in ALS and related disorders.

**Supplementary Information:**

The online version contains supplementary material available at 10.1007/s00018-025-05589-w.

## Introduction

Amyotrophic lateral sclerosis (ALS) and ALS associated with fronto temporal lobar degeneration (ALS-FTLD) are devastating neurodegenerative disorders characterized by progressive muscular paralysis due to the degeneration of upper and lower motor neurons in the primary motor cortex, corticospinal tract, brainstem and spinal cord. The deterioration of the patient’s conditions is irreversible and death occurs within 1–5 years after the onset for more than 70% of patients, often by respiratory failure [1, 2].

ALS and ALS-FTLD are proteinopathies, marked by the accumulation of misfolded proteins in the form of cytoplasmic inclusions in neuronal and glial cells. The TAR DNA binding protein of 43 kDa (TDP-43) represents the major component of pathological inclusions found in the brain and spinal cord of ALS and ALS-FTLD patients [3–5]. TDP-43 is a DNA/RNA-binding protein with several essential functions. In physiological situation, TDP-43 is mainly nuclear but shuttles from the nucleus to the cytoplasm. In nuclei, TDP-43 can drive or modulate key processes including transcription, splicing and RNA transport, whereas in the cytoplasm, it is involved in different mechanisms regulating miRNA processing, translation, mitochondrial functions, axonal transport or stress response pathways [6–9]. In pathological situations, TDP-43 loses its nuclear distribution and accumulates in cytoplasmic inclusions [3, 4, 9, 10]. In this context, diseased cells are characterized by the loss of TDP-43 functions in the nucleus and in the cytoplasm whereas, at the same time, TDP-43 aggregation causes gain of toxic functions, in particular, by sequestering RNAs and RNA binding proteins [11].

Over the past decade, various studies have demonstrated that misfolded TDP-43 from ALS and ALS-FTLD patient brains can induce TDP-43 aggregation in vitro, in cultured cells and in vivo in mouse models. This process occurs in a seed-dependent and self-templating manner, reminiscent of prion behavior [12–19].

Although growing evidences suggest that pathological TDP-43 inclusions can spread from cortical neuronal projections via axonal transport and synaptic contacts to the spinal cord and other regions of the brain through a prion-like model, the exact mechanisms of propagation and the nature and composition of the seeding activity remain unclear [20, 21]. Various modes of transmission have been proposed including cell–cell contacts via tunnelling nanotubes [22], or extracellular vesicles (exosomes and microvesicles) [13, 19, 22–24] and/or free aggregates [25]. Understanding the molecular and cellular mechanisms by which misfolded TDP-43 spreads is of utmost importance to identify potential therapeutical strategies that could block or slow down disease progression.

Recently, Lee et al*.,* (2016) reported a new unconventional secretion pathway called MAPS (for misfolding-associated protein secretion), that promotes the release of misfolded proteins like α-synuclein in the extracellular environment [26]. MAPS is orchestrated by the highly conserved ubiquitin-specific peptidase 19 (USP19), a member of the USP deubiquitinase family that is anchored to the cytosolic face of the endoplasmic reticulum (ER) through a transmembrane domain [26–28]. Overexpression of USP19 promotes its targeting to the ER membrane [29]. In this location, USP19 can intercept misfolded proteins destined for proteasomal degradation and redirect them to the secretory endosomal pathway for extracellular release [26, 28–31].

Although USP19-mediated MAPS has been well studied in the context of α-synuclein aggregation and secretion [26, 28, 30, 32, 33], its role in the secretion of ALS-associated pathological proteins, particularly misfolded TDP-43, has not been explored yet.

In this study, we report that overexpression of the catalytically active, ER-anchored USP19 in two distinct cellular models significantly enhances the secretion of free misfolded TDP-43 into the extracellular medium. This process occurs through an intracellular membrane trafficking pathway that involves early autophagic (phagophores and autophagosomes) and late endosomal/amphisomal compartments. Our investigations into the molecular and cellular mechanisms underlying this release reveals that several key proteins modulate USP19-mediated secretion of misfolded TDP-43. These include ATG7, ESCRT-0-HRS/HGS, RAB11A, RAB8A, RAB27A, VAMP7, and DNAJC5/CSPα.

In summary, this study reveals a new pathway by which misfolded TDP-43 can be released into the extracellular environment.

## Materials and methods

### Antibodies

The polyclonal anti-TDP-43 (12892–1-AP; WB 1: 2,000; 10782–2-AP; WB 1:2,000; IF 1:200; IEM 1:50), the monoclonal anti-TDP-43 (60019–2-Ig; IEM 1:50), the polyclonal anti-HA (51064–2-AP; WB 1:4,000; IF 1:100), the polyclonal anti-RAB27A (17817–1-AP; WB 1:2,000) and the USP19 polyclonal antibody (25768–1-AP; WB 1:1,000) were purchased from Proteintech. The anti-Flag (D6W5B) (#14793; WB 1:3,000; IF 1:200), -GAPDH (#2118; WB 1:10,000), -LC3B (#2775; WB 1:2,000; IF 1:100), -LC3B-E5Q2K (#83506; IF 1:200), -phosphor S6-ribosomal protein (Ser240/244) (#2215; WB 1:2,000), -RAB8A (D22D8) (#6975; WB 1:2,000), -RAB11A (D4F5) (#5589; WB 1:2,000), -S6 ribosomal protein (5G10) (#2217; WB 1:2,000), and -Ubiquitin conjugate HRP (#14049, WB 1: 2,000) were purchased from Cell Signaling Technology. The anti-Flag (NB600-344; IF 1:300), -GM130/GOLGA2 (NBP2-53420; IF 1:200) and -KDEL (NBP1-97469; IF 1:200) were purchased from Novus Biologicals. The anti-Flag M2 (F1804-50UG; IP 1:50), -GFP N-terminal (G1544; WB 1:2,000) and -HA 3F10 (11867423001, WB 1:2,000) were purchased from Sigma-Aldrich. The anti-SARS-CoV/SARS-CoV-2 (COVID-19) spike antibody 1A9 (GTX632604; IP 1:50) was purchased from GeneTex. The anti-CD63 (556019; WB 1:300 and 0.5% milk) and anti-CD81 (555675; WB 1:500 and 0.5% milk) were purchased from BD bioscience. The anti-CSP (154 003; WB 1:2,000) was purchased from synaptic system. The anti-HRS/HGS (ab155539; WB 1:1,000 and 5% milk) was purchased from Abcam. The anti-Myc (#13–2500 9E10; WB 1:1,000) was purchased from Invitrogen.

The secondary antibodies anti-mouse (1706516), anti-rabbit (1706515) (Bio-Rad Laboratories), the anti-guinea pig (106-035-003; Jackson ImmunoResearch) and the anti-rat (sc-2956; Santa Cruz) labeled with horseradish peroxidase (HRP) were used at 1:10,000. For immunofluorescence, the donkey anti-mouse Alexa Fluor 488 (A21202), and the donkey anti-goat Alexa Fluor 647 (A24447) were purchased from ThermoFischer. The donkey anti-rabbit Alexa Fluor 488 (A21206) was purchased from Molecular Probes. The donkey anti-rabbit Alexa Fluor Plus 555 (A32794) were purchased from Invitrogen. All these secondary antibodies were used at 1:1,000. For immunogold electron microscopy, the goat anti-mouse IgG (80.022-05) or anti-rabbit IgG (810.011-05) conjugated with 10 nm gold were used at 1:50 and purchased from Aurion. For immunohistochemistry, the avidin-biotinylated anti-rabbit IgG (BA-1000–1.5) was used at 1:1,000 and was purchased from Vector Laboratories.

### Plasmids

The mammalian expression constructs pRK-Flag-USP19 wild-type (USP19_WT_, Addgene #78597), USP19^1-1290^ (USP19_ΔTM_, Addgene #78579), USP19^1-493^ (USP19_Nter_, Addgene #78581), USP19_494-1318_ (USP19_Cter_, Addgene #78587) and USP19_C506S_ (Addgene #78584) were purchased from Addgene and were previously described [26]. The pCR3-uniTM (Invitrogen) empty control vector (CT) was previously described in [34].

The constructs pEGFPC1 encoding GFP-tagged TDP-43-ΔNLSΔ187-192 and -CTF-219-414 and the untagged pcDNA3-TDP-43-WT and K263E were provided by T.Nonaka [35]. The HA-tagged-TDP-43-K263E (in pDEST30) was provided by C.Shaw (London, UK) [36]. The Myc-tagged-TDP-43-K263E (in pcDNA3.1) was produced by GenScript.

The expression constructs pEGFPC3-GFP-tagged-VAMP7 wild-type (Addgene #42316), VAMP7-Longin (Addgene #42317), the pRFP-tagged-VAMP7-wild type and the pRFP-tagged-Longin were kindly provided by T.Galli (IPNP, Paris, France). The pMRXIP-GFP-tagged-YKT6 WT and Longin were kindly provided by N.Mizushima (Tokyo, Japan) [37].

The expression constructs 3xFLAG-CMV10 encoding flag-tagged-CSPα wild-type and the CSPα-S10A mutant were provided by T.Shirafuji (Kobe, Japan) [38].

The USP19 sgRNA1 and sgRNA2 used for USP19 knockout in HEK293T cells were provided by Yihong Ye (Addgene plasmids #78585 and #78586).

The pRK5-HA-Ubiquitin-WT was a gift from Ted Dawson (Addgene plasmid # 17608).

### Cell lines

The human embryonary kidney cell line (HEK293T) was purchased from the ATCC. HEK293T USP19 knockout cells were generated with CRISPR-Cas9 as previously described [26].

Cells were cultured in Dulbecco’s modified Eagle’s medium containing glutamax/pyruvate/glucose (DMEM) supplemented with 10% (v/v) of decomplemented fetal bovine serum (FBS) and 1% of penicillin/streptomycin (P/S). The Human neuroblastoma SH-SY5Y cell line was purchased from the ATCC and was cultured in DMEM/F12 supplemented with 10% of decomplemented FBS, 1% of P/S and 1% of MEM-non-essential amino acid (MEM-NEAA). Cell lines were maintained at 37 °C under 5% of CO_2_.

All cell culture media and reagents were purchased from Gibco-Thermo Fisher Scientific.

### Transient transfections

One day prior transfection HEK293T cells were plated at a density of 800,000 cells per well on 6-well plates in presence or absence of coverslips. Cells were transfected with 1–2 µg of total DNA using the FuGENE® HD transfection reagent (Promega) according to the manufacturer’s protocol. Alternatively, HEK293T cells were plated at a density of 4 million cells per 100 mm-dish. Cells were transfected with 10–20 µg of DNAs using the phosphate calcium method [39]. Cells and supernatant were recovered no more than 2 days after transfection for further analysis.

The SH-SY5Y cells were grown to 50% confluence in 6-well culture dishes for transient expression and then transfected with expression plasmids using XtreamGENE9 (Roche) according to the manufacturer’s instructions. Cells and supernatants were recovered no more than 3 days after transfection for further analysis.

### RNA interference

ATG7, DNAJC5/CSPα, HRS/HGS, RAB8A, RAB11A and RAB27A were downregulated using small interference RNA ON-TARGETplus SMARTpool (Dharmacon reagents, Horizon) human siDNAJC5/CSPα (L-024098-01-0005), siHRS (L-016835-00-0005), siRAB8A (L-003905-00-0005), siRAB11A (L-004726-00-0005), siRAB27A (L-004667-00-0005), siATG7 (L-020112-00-0005) or ON-TARGETplus non targeting pool (D-001810–10-05) as negative control. Transfection of the different siRNAs (control and gene of interest) was conducted using the Lipofectamine RNAi max reagent (Thermo Fischer Scientific) on day 1 on HEK293T cells in 6 well plates. Medium was changed on day 2, and cells were co-transfected with USP19-WT and TDP-43-K263E. Fresh complete medium (1.2 mL) was replaced on day 3 for an additional 16 h and conditioned media was collected and analyzed.

Knockdown efficiency was monitored by Western blotting using the antibodies directed against the respective targets.

### Drugs and compound

HEK293T cells co-expressing USP19-WT and TDP-43-K263E were treated overnight with the autophagic flux inhibitors Bafilomycin A1 and chloroquine at 5 nM in DMSO and 50 µM in water respectively or with the PI3K kinase inhibitors Spautin-1 and LY294002 at 1 µM and 50 µM in DMSO respectively. Conditioned media were collected, centrifuged at 12,000 rpm and analyzed by filter trap assay (FTA). All drugs were purchased from Sigma-Aldrich.

### Protein secretion experiments: filter trap assay (FTA)

To measure the secretion of misfolded TDP-43, collected conditioned media (full complete medium) were first centrifuged at 12,000 rpm for 5 min to remove cells and debris in suspension. The medium was mixed with 2X PBS 2% SDS (v/v) before being filtered through a nitrocellulose membrane (GE Healthcare Life Science, 0.22 µm), pre-wetted in 1X PBS 1% SDS, using Dot-blot™ apparatus (Bio-Rad Laboratories). After filtration, membranes were washed with 1X PBS containing 1% SDS and blocked in Tris-Buffered Saline Tween (TBS buffer; UP74004B, Interchim) containing 5% milk and 0.1% Tween (TBST) during 30 min before immunoblotting.

To visualize soluble misfolded TDP-43, identical volume of conditioned media collected before or after ultracentrifugation were dotted on a nitrocellulose membrane (Dot-blot™ apparatus Bio-Rad Laboratories) as previously depicted but in absence of SDS detergent to avoid protein solubilization. Membranes were dried and blocked in 5% milk TBST during 30 min before immunoblotting.

### Sucrose density gradient centrifugation

Conditioned medium was precleared at 12,000 rpm to eliminate cells and debris in suspension and then ultracentrifuged at 120,000×*g* for 75 min (120 kg pellet) at 4 °C in an SW32Ti rotor (Optima™ XE Ultracentrifuge, Beckman). The 120 kg pellet was directly analyzed by SDS-PAGE electrophoresis and Western blotting or resuspended in 500 µL 1X PBS supplemented with protease inhibitors and layered onto a 10–60% linear sucrose gradient. Sucrose gradient was ultracentrifuged for 19 h at 100,000×*g* at 4 °C in an SW41Ti rotor. Fractions (× 15) were recovered from the top of the gradient and densities were determined. Each fraction was diluted with reduced or non-reduced 5X sample buffer (312.5 mM Tris–HCl pH 6.8, 50% glycerol, 10% sodium dodecylsulfate (SDS), in presence or absence of 25% 2-mercaptethanol, bromophenol blue) and were analyzed by Western blotting for TDP-43 and extracellular vesicles markers respectively. Alternatively positive fractions were diluted in 1X PBS and re-ultracentrifuged for concentration and transmission electron microscopy analyses.

### Sarkosyl fractionation

Two to three days after transfection, cells were harvested and lysed in HB homogenization buffer (10 mM Tris–HCL pH 7.5, 0.8 M NaCl, 1 mM ethylene glycol bis β-aminoethyl ether-*N, N, N, N*-tetraacetic acid—EGTA) supplemented with 1% N-laurylsarcosine sodium salt (Sarkosyl), protease inhibitors and phosphatase inhibitors. Cell lysates were briefly sonicated (Bioruptor® Plus sonication, Diagenode) and then ultracentrifuged at 100,000x*g* during 30 min at room temperature in a TLA-100.3 rotor (Optima™ MAX-XP Ultracentrifuge, Beckman). The supernatant was recovered as Sarkosyl-soluble fraction (Sark-sol) and mixed with 5X sample buffer. The pellet was washed in HB buffer, resuspended in sample buffer, sonicated for solubilization and then used as the Sarkosyl-insoluble fraction (Sark-ins).

### Immunoprecipitation

Cells were lysed in radio-immunoprecipitation assay lysis buffer (RIPA R0278; Sigma Aldrich) containing protease (complete; Sigma-Aldrich) and phosphatase inhibitors (PhosphoStop; Roche). Samples were centrifuged twice at 800x*g* for 10 min and post-nuclear supernatants were recovered. Protein concentration was determined and 350–500 µg were precleared with Dynabeads Protein G (Thermo Fischer Scientific) for 1 h at 4 °C. Post-nuclear supernatants were recovered and incubated with beads coated with anti-Flag M2 antibody (Sigma-Aldrich, F1804-50UG), or anti-SARS-CoV-2 spike antibody 1A9 (GeneTex, GTX632604) as negative control or alternatively with an anti-Myc antibody overnight at 4 °C. Beads were recovered, washed with RIPA buffer and resuspended in 2X sample buffer. Samples were analyzed by SDS-PAGE electrophoresis and Western blotting using primary antibodies directed against Flag, Myc and/or HA tags or the anti-TDP-43.

### SDS-PAGE electrophoresis and immunoblotting

Whole cell lysates were prepared in 1X sample buffer supplemented with protease and phosphatase inhibitors cocktail. Each sample was loaded with the same volume or, alternatively, when necessary, protein concentration was determined with the Bradford reagent (Bio-Rad Laboratories) and 5–30 µg of proteins were loaded.

Samples (cell lysates, immunoprecipitates, Sark-sol and Sark-insol, fractions) were separated by 10–12% SDS-PAGE electrophoresis gels in presence of 2,2,2-trichloroethanol (TCE, T54801, Sigma-Aldrich) for stain free gel analyses using ChemiDoc™Touch imaging system (Bio-Rad Laboratories) analyzer. Gels were transferred to polyvinylidene difluoride (PVDF) membranes (Millipore, IPVH0001, 0.45 µm) by semi-dry electro transfer apparatus (Transblot® Turbo™, Bio-Rad Laboratories) in TOWBIN buffer (25 mM Tris, 192 mM glycine, 20% ethanol). Membranes were blocked with 5% milk TBST during 30 min. Membranes were probed with primary antibody in TBST overnight at 4 °C. After three washes with TBST, membranes were probed with HRP-conjugated secondary antibodies for 1 h at room temperature (RT) and washed. Immunodetection was conducted using the ECL™ reagent (Amersham) and revelation was carried using a ChemiDoc™ Touch imaging system. Quantification of immunoblots was done using the image lab software (Bio-Rad Laboratories). The sample loading was controlled using the anti-GAPDH antibody.

### Immunofluorescence and confocal microscopy imaging

HEK293T cells were grown for 24 h on 12-mm diameter coverslips coated with Poly-D-Lysin/Laminin (354087, Corning) and were transfected with the indicated DNAs/siRNAs as depicted above. After the indicated incubation time, cells were washed in 1X PBS, fixed with 4% paraformaldehyde (PFA) in 1X PBS at RT for 10 min, permeabilized with 0.2% Triton X-100 (Sigma-Aldrich) in 1X PBS for 5 min and blocked for 45 min with 1X PBS containing 2% bovine serum albumin (BSA, Sigma-Aldrich) at RT. Staining were performed overnight at 4 °C with the indicated primary antibody in the saturation buffer. After washing with 1X PBS, secondary antibodies conjugated with Alexa-Fluor 488 or 555 or 647 were applied for 1 h at RT. After washing, nuclear DNA was subsequently counterstained with DAPI (D9542; Sigma-Aldrich) for 5 min at RT. Finally, the washed coverslips were mounted with FluorSave (Millipore). Images were acquired using the confocal microscope Zeiss LSM-880 (CIQLE, platform from the Faculty of Medicine of Lyon, France) and analyzed using the Fiji/ImageJ software.

### Transmission electron microscopy (TEM) of co-transfected cells

Transfected cells were fixed with 2% glutaraldehyde (EMS) in 0.1 M sodium cacodylate (pH7.4) buffer at room temperature for 30 min. After washing three times in 0.2 M sodium cacodylate buffer, cell cultures were post-fixed with 1% aqueous osmium tetroxide (EMS) at room temperature for 1 h and dehydrated in a graded series of ethanol at room temperature and embedded in Epon. After polymerization, ultrathin Sections (100 nm) were cut on a UC7 (Leica) ultramicrotome and collected on 200 mesh grids. Sections were stained with uranyl acetate and lead citrate before observation on a Jeol 1400JEM (Tokyo, Japan) transmission electron microscope equipped with a Orius 1000 camera and Digital Micrograph.

For immunolabeling, cells were fixed in 4% paraformaldehyde complemented with 0.2% glutaraldehyde for 1 h at 4 °C. Cells were washed three times in cacodylate 0.2 M saccharose 0.4 M for 15 min at 4 °C, dehydrated though a series washes with 30, 50, 70% ethanol maintained at 4 °C for 5 min, infiltrated with London Resin White (LRWhite, EMS, France) using 1:1 LRWhite and 4 °C absolute ethanol for 60 min followed by pure LRWhite at 4 °C for three periods of 60 min each, then embedded in pure LRWhite in gelatine capsules for polymerization at 50 °C for 48 h. Ultrathin Sections (100 nm thick) were cut on a UC7 (Leica) ultra-microtome, mounted on 200 mesh nickel grids and stabilized for 1 day at RT. Immunogold labelling was performed by flotation the grids on drops of reactive media. Non-specific sites were coated with 1% BSA and 1% normal goat serum (NGS) in 50 mM Tris–HCl, pH 7.4 for 20 min at RT. Thereafter, incubation was carried out overnight at 4 °C in wet chamber with primary antibody anti–TDP-43 (human specific) monoclonal antibody (ProteinTech, 60019–2-Ig) diluted at 1:50 in 1% BSA, 50 mM Tris–HCl, pH 7.4. Sections were successively washed three times in 50 mM Tris–HCl, pH 7.4 and pH 8.2 at RT. They were incubated in a wet chamber for 45 min at RT in 1% BSA, 50 mM Tris–HCl, pH 8.2, labeled with gold conjugated secondary antibody (Gold anti mouse 10 nm-Aurion). Sections were successively washed three times in 50 mM Tris–HCl pH 8.2 and pH 7.4 and three times with filtrated distilled water. The immunocomplex was fixed by a wash in glutaraldehyde 4% for 3 min. Sections were stained with 0.5% uranyl acetate in ethanol 50% for 5 min in darkness and observed with a transmission electron as describe above.

### Electron microscopy and immunogold analyses of sucrose density fractions

For analysis of sucrose fractions, the positive sucrose fractions were pooled concentrated at 120,000x*g* for 75 min at 4 °C in SW41Ti rotor. The 120,000x*g* pellets were gently resuspended in 50 µL PBS and fixed with the added of 50 µL mixture of 4% paraformaldehyde and 0.065% glutaraldehyde in PBS at 4 °C. Suspensions were absorbed on 200 Mesh Nickel grids coated with formvar-C for 10 min at RT. Immunogold labelling was performed the next day by flotation the grids on drops of reactive media. Unspecific sites were coated with 1% BSA in 50 mM Tris–HCl, pH7.4 for 10 min at RT. The incubation was carried 2 h at RT in wet chamber with primary antibody anti–TDP-43 (human specific) monoclonal antibody (ProteinTech, 60019–2-Ig) or TDP-43 polyclonal antibody (ProteinTech, 10782–2-AP) diluted at 1:50 in 1% BSA, 50 mM Tris–HCl, pH7.4. They were successively washed in 50 mM Tris–HCl, pH7.4 and pH8.2 at RT. Then, grids were incubated in a wet chamber in 1% BSA, 50 mM Tris–HCl pH8.2 for 10 min at RT, and labelled with 10 nm gold conjugated goat anti-mouse IgG or anti-rabbit IgG (Aurion) diluted 1:50 in 1% BSA, 50 mM Tris–HCl pH8.2 for 45 min. Grids were successively washed in 50 mM Tris–HCl pH8.2 and pH7.4. Then, immunocomplex was fixed with glutaraldehyde 4% diluted in 50 mM Tris–HCl pH7.4 for 2 min. Grids were contrasted and embedded in a mixture of 4% uranyl acetate and 2% methylcellulose for 10 min in the dark before observation on a transmission electron microscope as describe above.

### RNA-seq

Total RNA from HEK293T HA-TDP-43-K263E and Flag-USP19-WT or ΔTM co-expressing cells was extracted from three independent transfected cells. The RNA-seq library preparation was done with 150 ng of input RNA using the Illumina TruSeq Stranded mRNA Library Prep Kit. Paired-end RNA-seq were performed with Illumina NextSeq sequencing instrument (Helixio, Clermont-Ferrand, France). All statistical analyses were performed with the statistics software R (version 3.2.3; available from:https://www.r-project.org) and R packages developed by BioConductor project (available from: https://www.bioconductor.org). Analyses of data generated by the RNA-seq (GO pathway enrichment analyses, volcano and bubble/dot plots) were carried out using the open online platform SR-PLOT web server (https://www.bioinformatics.com.cn/en) [40].

### Statistical analysis

Data are shown as mean ± standard error of the mean (SEM). Data were analyzed in GraphPad Prism (version 9.0.0 for MacOS, GraphPad Software, San Diego, www.graphpad.com). A p-value below 0.05 was considered statistically significant.

## Results

### ER-associated USP19 promotes misfolded TDP-43 secretion

**Fig. 1:**
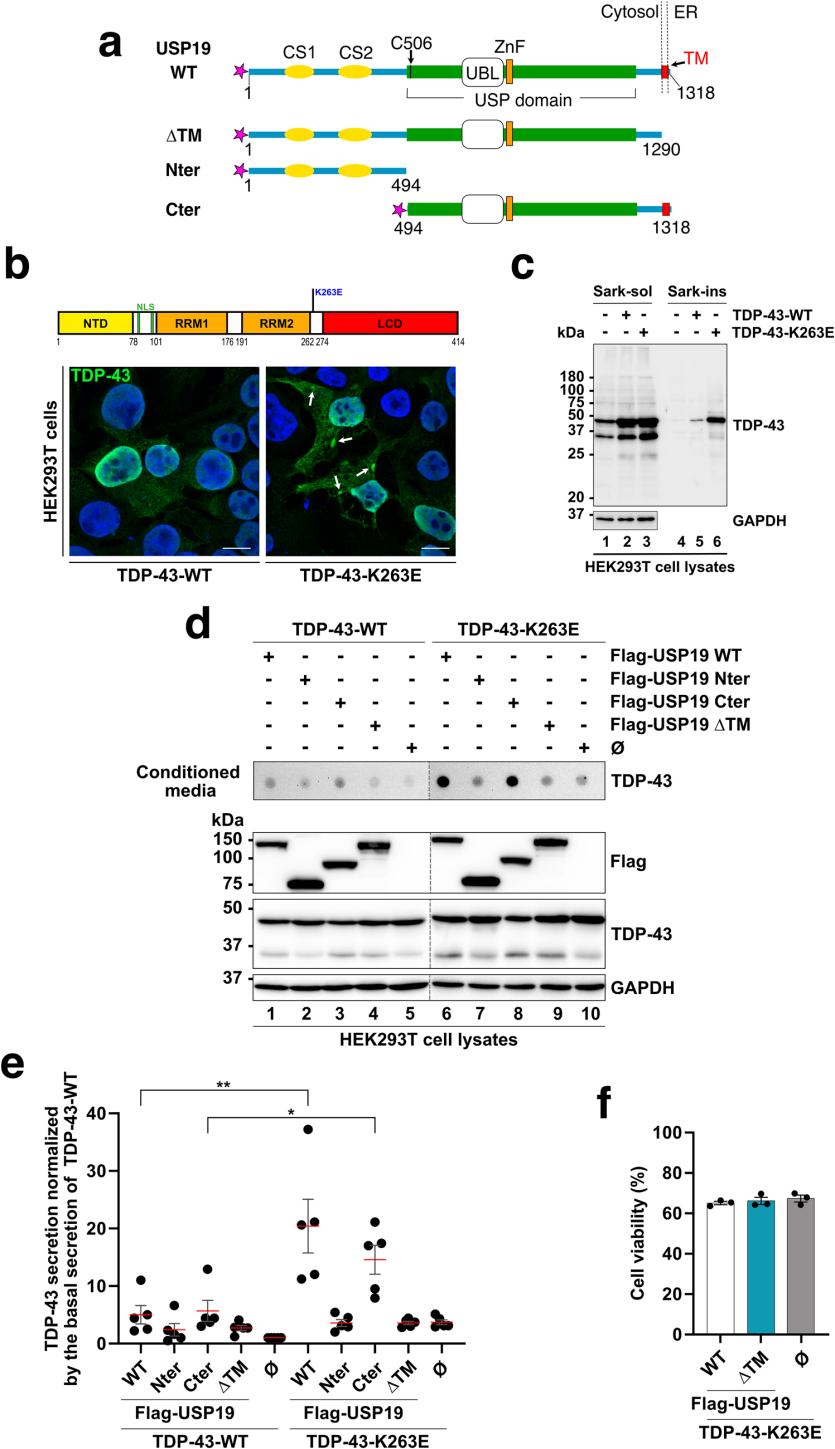
The ER-anchored USP19 promotes the secretion of misfolded TDP-43 in the HEK293T cellular model. **a** Schematic representation of Flag-USP19 constructs used in this study. The C506 residue represents an essential amino acid residue necessary for the ubiquitin peptidase catalytic activity. *CS 1&2* CHORD-containing proteins and STG1, *UBL* ubiquitin-like, *USP* ubiquitin-specific peptidase, *TM* transmembrane domain, *ZnF* zinc finger, *ΔTM* deleted for the transmembrane domain. The pink star represents the amino terminal Flag tag. **b** Schematic representation of the full-length TDP-43 (upper panel). The K263E variant is highlighted in blue. *LCD* Low Complexity Domain, *NLS* nuclear localization sequences, *NTD* N-terminal domain, *RRM1&2* RNA recognition motif 1&2. HEK293T cells were transfected with TDP-43-WT or TDP-43-K263E encoding constructs and were visualized by immunofluorescence using anti-TDP-43 antibody. Blue signal corresponds to DAPI for nuclei and green signal to TDP-43. Scale bar is 10 µm. **c** Immunoblotting of sarkosyl soluble supernatant (Sark-sol) and sarkosyl insoluble pellet (Sark-ins) fractions isolated from control HEK293T cells (lanes 1 and 4), or cells expressing TDP-43-WT (lanes 2 and 5) or TDP-43-K263E (lanes 3 and 6) using antibodies directed against TDP-43 and GAPDH as loading control. **d** Evaluation of misfolded TDP-43 secretion by filter trap assay (FTA). Presence of misfolded TDP-43 in conditioned media from HEK293T cells overexpressing TDP-43-WT and TDP-43-K263E and the different Flag-USP19 or the empty vector (negative control) was monitored by FTA. Upper panel (secretion/conditioned media): nitrocellulose membranes were probed with an antibody directed against TDP-43. Lower panel (cell expression): Western blotting of cell lysates from co-expressing cells using anti-Flag (for USP19), -TDP-43 and -GAPDH antibodies for loading control. **e** Quantification of secreted TDP-43 upon USP19 expression. Data represent mean ± SEM, *n* = 5 experiments. Significance was assessed by a Mann–Whitney U test (**p < *0.001*). **f** USP19-WT or USP19-ΔTM and TDP-43-K263E overexpression does not affect plasma membrane permeability and cell viability. HEK293T cells transfected with the indicated plasmids were stained with trypan blue and counted. Data represent mean ± SEM,* n* = 3 experiments

Various studies have shown that overexpression of the ER-associated USP19 ubiquitin peptidase promotes unconventional secretion of misfolded proteins such as α-synuclein, into the extracellular medium via the MAPS pathway [26, 28, 30]. Conversely, no secretion of misfolded proteins was observed when a cytosolic form of USP19, deleted for its transmembrane domain necessary for ER-anchoring (USP19-ΔTM; see Fig. 1a), was overexpressed. These data indicate the crucial role of its association to the ER membrane in the secretion process [26, 28]. Interestingly, Xu et al., (2018) also observed that co-expression of USP19 with wild type TDP-43, barely increased the amount of TDP-43 in the extracellular medium [28]. These data prompted us to investigate the possibility that ER-USP19 could specifically promote the secretion of misfolded TDP-43 mutants prone to aggregation. In this context, various combinations of WT and mutant TDP-43 and USP19 were overexpressed in HEK293T cells. We first overexpressed untagged-TDP-43-WT and the familial TDP-43-K263E ALS variant [36] (Fig. 1b). Immunofluorescence experiments using anti-TDP-43 antibodies confirmed that wild type TDP-43 was predominantly expressed in the nucleus with a slight cytoplasmic distribution, whereas the K263E variant was distributed both in the nucleus and in the cytoplasm where large inclusion bodies could be observed (see white arrows in Fig. 1b). The presence of TDP-43 aggregates was confirmed by Western blotting after sarkosyl fractionation: a significant amount of TDP-43-K263E mutant was present into the insoluble fractions (Sark-ins) whereas only a faint signal was observed with TDP-43-WT (compare lanes 5 and 6 in Fig. 1c).

As expected, Flag-USP19-WT strongly accumulated in the ER as evidenced by colocalization with the KDEL ER-marker. In contrast, the USP19-ΔTM mutant (Fig. 1a), lacking the transmembrane domain necessary for ER-anchoring did not colocalize with KDEL labeling (see Fig. S1a, b). Neither USP19-WT nor USP19-ΔTM colocalized with the Golgi apparatus marker GM130 (Fig. S1c, d).

Based on these data, we next investigated the impact of flagged USP19-WT and ΔTM on TDP-43-WT secretion in co-expressing cells. To evaluate TDP-43 secretion, conditioned media were analyzed by filter trap assay (FTA) in presence of 1% SDS detergent to specifically detect insoluble TDP-43 aggregates. The results presented in Fig. 1d, e showed that USP19-WT only slightly promoted the secretion of TDP-43-WT aggregates compared to the USP19-ΔTM, corroborating previously published data [28]. Conversely, when co-expressed with the pathological TDP-43-K263E (Fig. 1b), USP19-WT, but not the USP19-ΔTM, significantly increased the secretion of aggregated TDP-43 (Fig. 1d, e). Similar results were obtained when HA-Tagged TDP-43-K263E was co-expressed with USP19-WT in the presence (Fig. S2a-c) or absence of endogenous USP19 expression (Fig. S2d, e).

Interestingly, Xu et al*.,* (2018) observed that the C-terminal region of USP19 encompassing amino acid residues 494–1318 (USP19^494−1318^; see Fig. 1a), which include the Ubiquitin peptidase activity and the ER-anchoring transmembrane domain, was sufficient to enhance the secretion of the misfolded proteins including α-synuclein [28]. In contrast, overexpression of the N-terminal part of USP19 (USP19^1−493^; Fig. 1a) did not induce secretion [26]. These data prompted us to evaluate the impact of both truncated forms of USP19 on TDP-43-WT and TDP-43-K263E secretion. The data presented in Fig. 1d,e, confirmed that USP19^494−1318^ promoted the secretion of misfolded TDP-43-WT and K263E, while the N-terminal USP19^1−493^ did not enhance the release. This indicates that the 494–1318 domain of USP19 contains the essential functional properties for misfolded TDP-43 secretion.

To ensure that the observed secretion of misfolded TDP-43 was not simply the reflect of cell death potentially induced by the combined expression of USP19-WT and TDP-43-K263E, we assessed cell viability using the trypan blue exclusion method. The data presented in Fig. 1f and S2c revealed comparable levels of viable cells across the conditions. These findings, indicate that the secretion of misfolded TDP-43 was a specific cellular process rather than a byproduct of increased cell mortality.

Increased secretion of misfolded TDP-43 in the presence of USP19-WT or the C-terminus USP19^494−1318^ was confirmed with the cytosolic GFP-tagged TDP-43-ΔNLSΔ187-192 [35] mutant (Fig. S3a-c). Interestingly, when the same experiments were conducted with the C-terminus prion-like domain (GFP-TDP-43-CTF^219−414^) prone to high aggregation [35] (see Fig. S3a), neither USP19-WT nor its mutants stimulated TDP-43 secretion (Fig. S3d).

To validate our findings in a neuron-like model, we investigated the impact of USP19-WT on TDP-43-K263E secretion in the human neuroblastoma SH-SY5Y cell line. The data presented in Fig. S4a, b confirmed that overexpression of USP19-WT promotes the secretion of the misfolded TDP-43-K263E in this neuronal cellular model.

Overall, the data correlate well with those previously published on other prion-like proteins and indicate that the ER-anchored USP19 can promote the secretion of ALS TDP-43 misfolded mutants in different cellular models.

### USP19 ubiquitin peptidase activity is essential for TDP-43 secretion

**Fig. 2:**
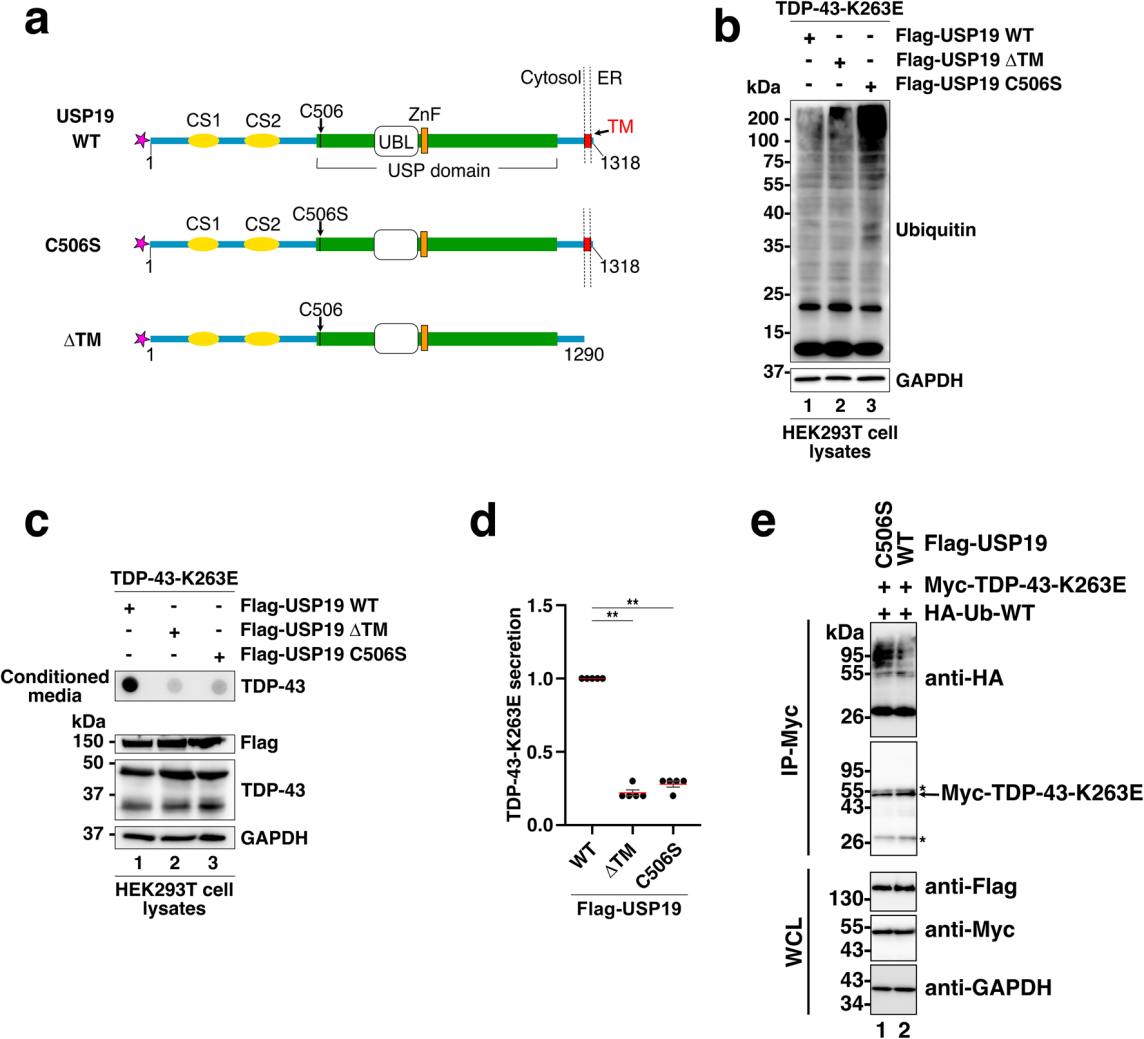
The ubiquitin peptidase activity is essential for TDP-43-K263E secretion in USP19 context. **a** Schematic representation of Flag-USP19 constructs. The USP19-C506S corresponds to an ER-anchored but ubiquitin peptidase catalytically inactive mutant. **b** Ubiquitination level in cell lysates of co-expressing cells. Immunoblotting of cell lysates from TDP-43-K263E and the indicated Flag-USP19 co-expressing cell using antibodies directed against Ubiquitin or GAPDH as loading control. **c** Evaluation of misfolded TDP-43 secretion by FTA. Presence of misfolded TDP-43 in conditioned media from HEK293T cells overexpressing the TDP-43-K263E, the WT, the ΔTM or the C506S Flag-USP19, was monitored by FTA. Upper panel (secretion/conditioned media): nitrocellulose membranes were probed with an antibody directed against TDP-43. Lower panel (cell expression): Western blotting of cell lysates from co-expressing cells using anti-Flag (for USP19), -TDP-43 and -GAPDH antibodies for loading control. **d** Quantification of secreted TDP-43-K263E upon USP19s expression. Data represent mean ± SEM, *n* = 5 experiments. Significance was assessed by a Mann–Whitney *U* test (**p < 0.001). **e** TDP-43-K263E deubiquitination is promoted by USP19-WT. Lysates from HEK293T cells co-expressing Myc-TDP-43-K263E and HA-Ubiquitin-WT with Flag-USP19-C506S (ubiquitin peptidase deficient mutant; lane 1) or Flag-USP19-WT (lane 2) were immunoprecipitated with anti-Myc and immunoblotted with anti-HA and anti-Myc. Whole cell lysates (WCL; 5 μg/input) from co-expressing cells were immunoblotted using anti-Myc, anti-Flag and anti-GAPDH for loading control. Asterisks correspond to heavy and light chains of immunoglobulins used during the immunoprecipitation

Ubiquitinated and hyperphosphorylated TDP-43 was identified as a primary constituent of the mislocalized and insoluble cytoplasmic inclusions in the ALS and ALS-FTLD affected brains [3, 4]. Recently, a growing body of evidence indicated that ubiquitinating and deubiquitinating pathways are critically engaged in the fate decision of aberrant or pathological TDP-43 proteins [41]. The data presented above revealed that USP19-WT promotes the secretion of misfolded TDP-43 but the mechanisms by which USP19-WT induces this secretion are still unresolved. In this context, we first evaluated the involvement of the USP19 ubiquitin peptidase activity on the misfolded TDP-43-K263E secretion. For this purpose, the ubiquitin peptidase catalytically inactive USP19-C506S mutant [28] was co-expressed with the TDP-43-K263E mutant in HEK293T cells (Fig. 2a). Western blotting on whole cell extracts showed that expression of the USP19-C506S mutant increased the levels of ubiquitinated proteins compared to USP19-WT and USP19-ΔTM (compare lanes 1&2 with lane 3 in Fig. 2b). Analysis of the culture medium by FTA revealed that the increase in protein ubiquitination was associated with a loss of TDP-43-K263E secretion, suggesting that beyond its association with the ER membrane, USP19 ubiquitin peptidase activity is crucial for the secretion of misfolded TDP-43 (Fig. 2c, d).

To determine if misfolded TDP-43-K263E is ubiquitinated in our cellular system and requires to be deubiquitinated by USP19-WT for its secretion, co-immunoprecipitation experiments were conducted on lysates from HEK239T cells co-expressing a Myc-tagged-TDP-43-K263E, an HA-tagged-Ubiquitin-WT, and either the Flag-USP19-WT (promoting TDP-43 secretion) or the deubiquitinase-deficient Flag-USP19-C506S (no TDP-43 secretion context). Myc-TDP-43-K263E was immunoprecipitated using an anti-Myc antibody, and the immunoprecipitate was analyzed by Western blotting using an anti-HA antibody to detect the linked HA-Ubiquitin-WT. The data presented in Fig. 2e confirmed the ubiquitination of Myc-TDP-43-K263E when the Flag-USP19-C06S mutant was expressed (Lane 1), compared to lower ubiquitination levels when Flag-USP19-WT was expressed (Lane 2).

Overall, these findings support the conclusion that TDP-43-K263E deubiquitination and secretion is promoted by the USP19-WT expression.

### USP19 overexpression promotes the secretion of soluble and free TDP-43 aggregates

**Fig. 3:**
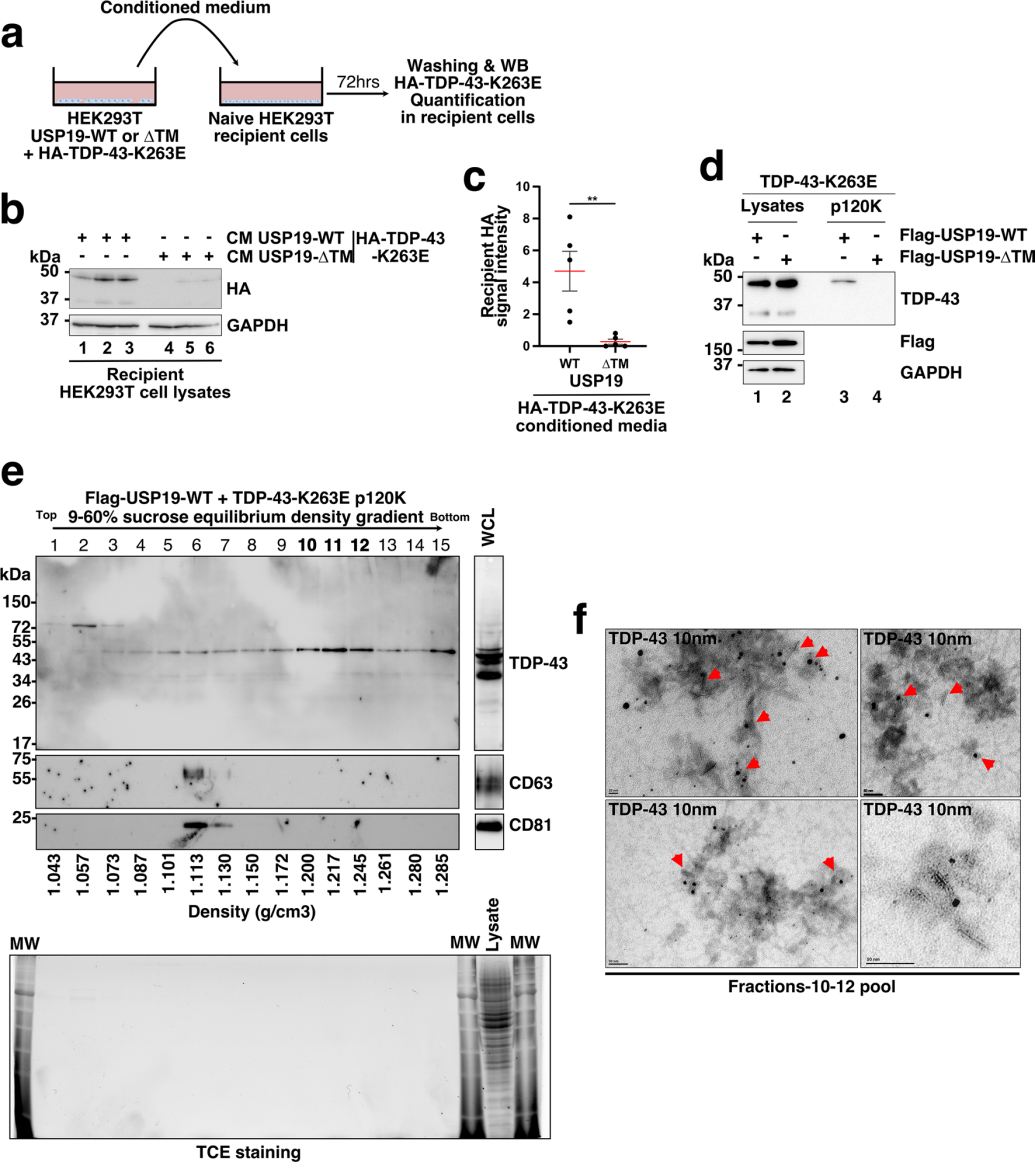
The ER-anchored USP19 promotes the secretion of free TDP-43-K263E fibrils in the conditioned medium. **a** Transmission of misfolded HA-TDP-43-K263E to naïve HEK293T cells. Naive HEK293T cells were cocultured with conditioned media from HEK293T co-expressing cells Flag-USP19-WT or − ΔTM with HA-TDP-43-K263E during 72 h. **b** After 3 days, cells were washed and analyzed by Western blotting using anti-HA and anti-GAPDH as loading control. **c** Quantification of n = 5 independent experiments. Significance was assessed by a Mann–Whitney U test (**p < 0.001). **d** Aggregated TDP-43 levels in conditioned media from HEK293T cells overexpressing TDP-43-K263E and the Flag-USP19 WT and ΔTM were precleared to eliminate cellular debris and ultracentrifuged at 120,000 g pellet (p120K pellet) and analyzed by immunoblotting (lanes 3 and 4) using antibody directed against TDP-43. Cellular lysates (lanes 1 and 2) were analyzed by Western blotting and probed by antibodies directed against TDP-43, Flag and GAPDH (loading control). **e** Sucrose equilibrium density gradient fractionation of conditioned medium. The p120K pellet was fractionated through a linear sucrose equilibrium density gradient 9–60% and fractions (× 15), recovered from the top of the gradient, were analyzed by Western blotting using anti-TDP-43 or anti-CD81 and anti-CD63 antibodies for extracellular vesicles markers. Fraction density values (g/cm^3^) are depicted at the bottom panel. Whole cell lysate (WCL) of co-expressing cells was immunoblotted in parallel using the anti-TDP-43, anti-CD63 and anti-CD81. The bottom panel corresponds to the TCE staining of the SDS-PAGE gel. **f** Immunogold electron microscopy (IEM) of TDP-43-K263E positive fractions. Positive fractions (10–12) containing the TDP-43-K263E were pooled and analyzed by IEM using anti-TDP-43 labelled with a secondary antibody coupled with 10 nm gold particle. Amorphous and fibrillar structures were labelled (red arrows). Scale bars are 20 and 50 nm

Many studies have suggested that misfolded TDP-43 can be transmitted from cell to cell in a prion-like manner and contributes to the spreading of the pathology in different region of the central nervous system [13, 15, 16, 18–20]. Understanding the molecular and cellular mechanisms involved in the secretion as well as the transmission of pathological TDP-43 from cell to cell is of utmost importance to identify potential therapeutical targets. In this context, we tested whether USP19 overexpression can contribute to the cell–cell transmission of misfolded-TDP-43. For this purpose, conditioned medium from donor HEK293T cells co-expressing USP19-WT and HA-TDP-43-K263E were added to naïve HEK293T cells as previously depicted [42]. The data presented in Fig. 3a-c revealed that treatment of naïve HEK293T recipient cells with conditioned media from HEK293T donor cells co-expressing USP19-WT and HA-TDP-43-K263E showed a strong HA-TDP-43-K263E signal in recipient cells whereas only a very faint signal HA-TDP-43 was detected when the USP19–ΔTM mutant was expressed instead of USP19-WT in donor cells (Fig. 3b, c). These data suggest that USP19-WT overexpression may play a role in the intercellular transmission of secreted misfolded TDP-43 (Fig. 3a-c).

Today, it is currently accepted that pathological TDP-43 can be released into the extracellular environment through various pathways, including extracellular vesicles (exosomes and/or microvesicles) [13, 19, 22–24] and/or as free aggregates [25]. To characterize in which form TDP-43-K263E was secreted upon USP19-WT overexpression, conditioned media from cells co-expressing USP19-WT and TDP-43-K263E were collected, centrifuged to remove cellular debris and ultracentrifuged at 120,000x*g* to pellet vesicles and protein aggregates (referred to as p120K). Secreted misfolded TDP-43 was detected in the p120K pellet, but not in the USP19–ΔTM context (Fig. 3d), confirming the previous FTA experimental results.

The p120K pellet was then fractionated through a linear 9–60% sucrose density gradients. Fifteen fractions were collected from the top, and analyzed by immunoblotting using antibodies against TDP-43 and the CD63 and CD81 extracellular vesicles (EVs) markers (Fig. 3e). While a faint TDP-43 signal was observed in the CD63 and CD81 positive fractions 6 and 7, the majority of TDP-43 was detected in a second peak, in fractions of higher densities (fractions 10 to 12; Fig. 3e). No TDP-43 signal was detected in any gradient fractions of the USP19–ΔTM context, although CD63 and CD81 EV markers were well detected (Fig. S5).

Immunogold electron microscopy (IEM) performed on TDP-43 positive fractions 10–12 revealed small fibrillar aggregated structures labeled with anti-TDP-43 and a secondary antibody coupled with 10 nm gold particles (indicated by red arrowheads in Fig. 3f). Similar observations were made when the GFP-TDP-43-ΔNLSΔ187-192 mutant was co-expressed instead of TDP-43-K263E with USP19-WT (Fig. S3e, f).

Given that MAPS pathway has been shown to enhance the secretion of soluble misfolded proteins into the extracellular space [26, 28], we next investigated if soluble TDP-43 could be detected in the conditioned medium after ultracentrifugation. To address this point, similar experiments were carried out as depicted above using HEK293T cells co-expressing Flag-USP19-WT and HA-TDP-43-K263E. The conditioned medium was collected and divided, with one third set aside as non-ultracentrifuged and the remainder subjected to ultracentrifugation at 120,000xg as previously depicted. Analysis of the p120K pellets confirmed the presence of TDP-43 aggregates when USP19-WT was expressed but not in presence of USP19-ΔTM (Fig. S6a). We then compared the ultracentrifuged supernatant with the non-ultracentrifuged sample using dot-blot assay without SDS detergent treatment to detect if soluble TDP-43 was secreted. The data presented in Fig. S6b, c revealed the presence of soluble TDP-43 in the resulting ultracentrifuged supernatant albeit at lower levels compared to non-ultracentrifuged condition. As anticipated, little or no TDP-43 signal was observed when USP19-ΔTM was expressed.

In conclusion, these findings indicate that USP19-WT expression promotes the secretion of both soluble and aggregated forms of misfolded TDP-43.

### TDP-43 is associated with USP19 and the ER compartments and is engulfed in different intracellular compartments.

**Fig. 4:**
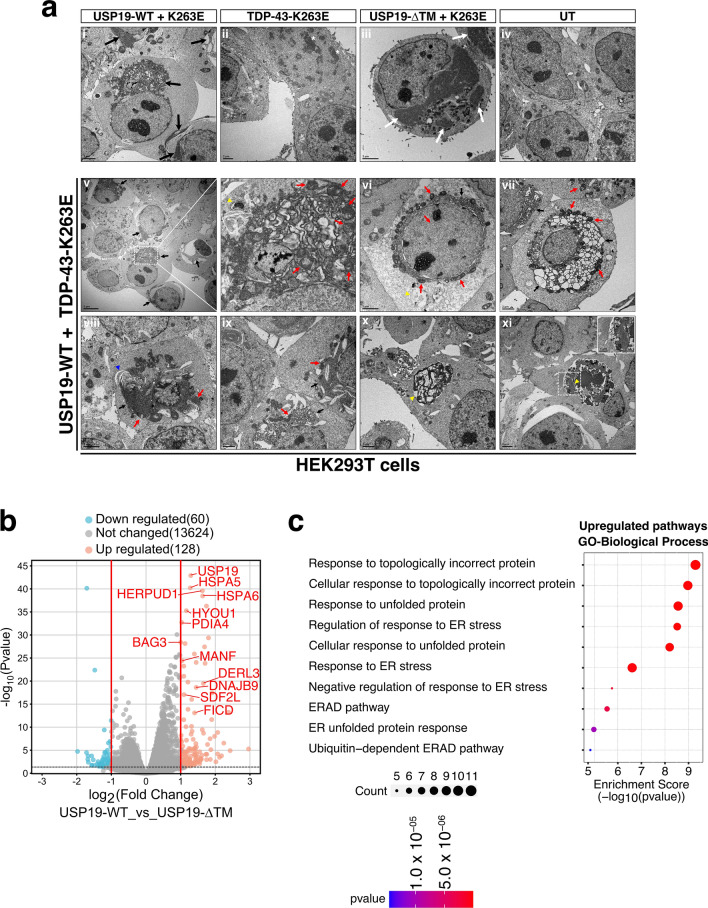
Transmission electron microscopy (TEM) and RNA sequencing analyses point out ER alterations in TDP-43-K263E and USP19-WT co-expressing cells. **a** Ultrastructural analyses. TEM of TDP-43-K263E + UPS19-WT (panel i); TDP-43-K263E (panel ii); TDP-43-K263E + USP19-ΔTM (panel iii) and untransfected cells (UT; panel iv as negative control). Black arrows indicate dilated endoplasmic reticulum (ER) accumulation (panels i and v; v-right corresponds to a higher magnification of panel v). White asterisk indicates cytoplasmic aggregates (panel ii). White arrows indicate compact electron dense structures (CEDS; panel iii). Red arrows indicate mitochondria in close contacts with ER (panels v-right to vii). Yellow arrowheads indicate autophagic/endosomal compartments containing ER membrane and dense amorphous structures. (panels v-right, x and xi). Scale bars are 2 and 5 μm. Dotted squares correspond to higher magnification **b** Volcano plot of differentially expressed genes between TDP-43-K263E/USP19-WT versus TDP-43-K263E/USP19-ΔTM co-expressing cells. Red dots represents upregulated genes (P < 0.05 and Log2FC > 1), blue dots represents downregulated genes (P < 0.05 and Log2FC < -1), and grey dots represent genes that were not differentially expressed. **c** Bubble plot of the GO Biological process pathway enrichment analysis. The horizontal axis represents the enrichment score (-log10(p-value)), while the vertical axis represents the enriched pathway name. The color scale indicates different thresholds of the p-value, and size of the bubble indicates the number of genes corresponding to each pathway

To further characterize the cellular mechanisms involved in USP19-dependant trafficking and secretion of TDP-43-K263E, transmission electron microscopy (TEM) experiments were conducted. HEK293T cells co-transfected with both TDP-43-K263E and USP19-WT were compared to cells expressing TDP-43-K263E alone, TDP-43-K263E with USP19-ΔTM, or untransfected cells as negative control. Notably, cells co-expressing TDP-43-K263E and USP19-WT exhibited a significant accumulation of dilated ER (indicated by black arrows in panels i and v-ix in Fig. 4a). This observation correlated with the strong KDEL signal observed in Fig. S1a. No such ER accumulation was observed in the other conditions.

RNA sequencing analyses performed on HEK293T cells co-expressing USP19-WT and TDP-43-K263E versus USP19-ΔTM and TDP-43-K263E revealed upregulated genes in USP19-WT co-expressing cells primarily associated with biological process linked to the ER including responses to topologically incorrect proteins, unfolded protein response, and, endoplasmic reticulum stress (Fig. 4b, c).

TEM analyses also revealed the presence of typical phagophore structures reminding those previously depicted in [43] (see blue arrowhead in Fig. 4a panel viii) and numerous intracellular compartments, containing engulfed parts of the endoplasmic reticulum, filamentous and amorphous dense structures within their lumen (see yellow arrowheads in Fig. 4a panels v, vi, x and xi). Mitochondria were also observed in close contact with the accumulated ER (see red arrows in Fig. 4a panels v to ix).

To better characterize these compartments, we initiated confocal immunofluorescence microscopy experiments on HEK293T cells co-expressing Flag-WT-USP19 and HA-TDP-43-K263E using anti-Flag (to detect USP19), anti-HA (to detect the mutant TDP-43) and anti-LC3 (to visualize endogenous LC3 positive compartments such as phagophores, autophagosomes or amphisomes). Three kinds of structures were observed: the first one corresponds to a compact structure where small inclusions/punctuated fine scattered patterns of TDP-43 are embedded and colocalized with USP19 and LC3 (see arrow and magnification in Fig. S7a,b). The second structure corresponds to TDP-43 punctuated fine scattered patterns surrounded by LC3, suggesting that TDP-43 is engulfed in an LC3-positive compartment (see arrow and magnification in Fig. S7c). The last one is an hybrid structure, where TDP-43 aggregates were embedded with LC3 and USP19 and surrounded by LC3 (see arrow and magnification in Fig. S7d). In conclusion, this set of data, correlated with the EM investigations, suggests that TDP-43, is detected with LC3 and/or USP19 positive intracellular structures reminding autophagic compartments.

Interestingly, compact electron dense structures (CEDS) were visible in TDP-43-K263E expressing cells but not in non-transfected cells (white asterisks in Fig. 4a panel ii), probably corresponding to TDP-43 aggregates. Bigger CEDS were also observed when TDP-43-K263E was co-expressed with USP19-ΔTM (white arrows in panel iii of Fig. 4a). These CEDS do probably not correspond to ER structures or USP19-ΔTM aggregates, since KDEL or Flag signal accumulations were not detected in cells co-expressing TDP-43-K263E and USP19-ΔTM (Fig. S1a, b).

**Fig. 5:**
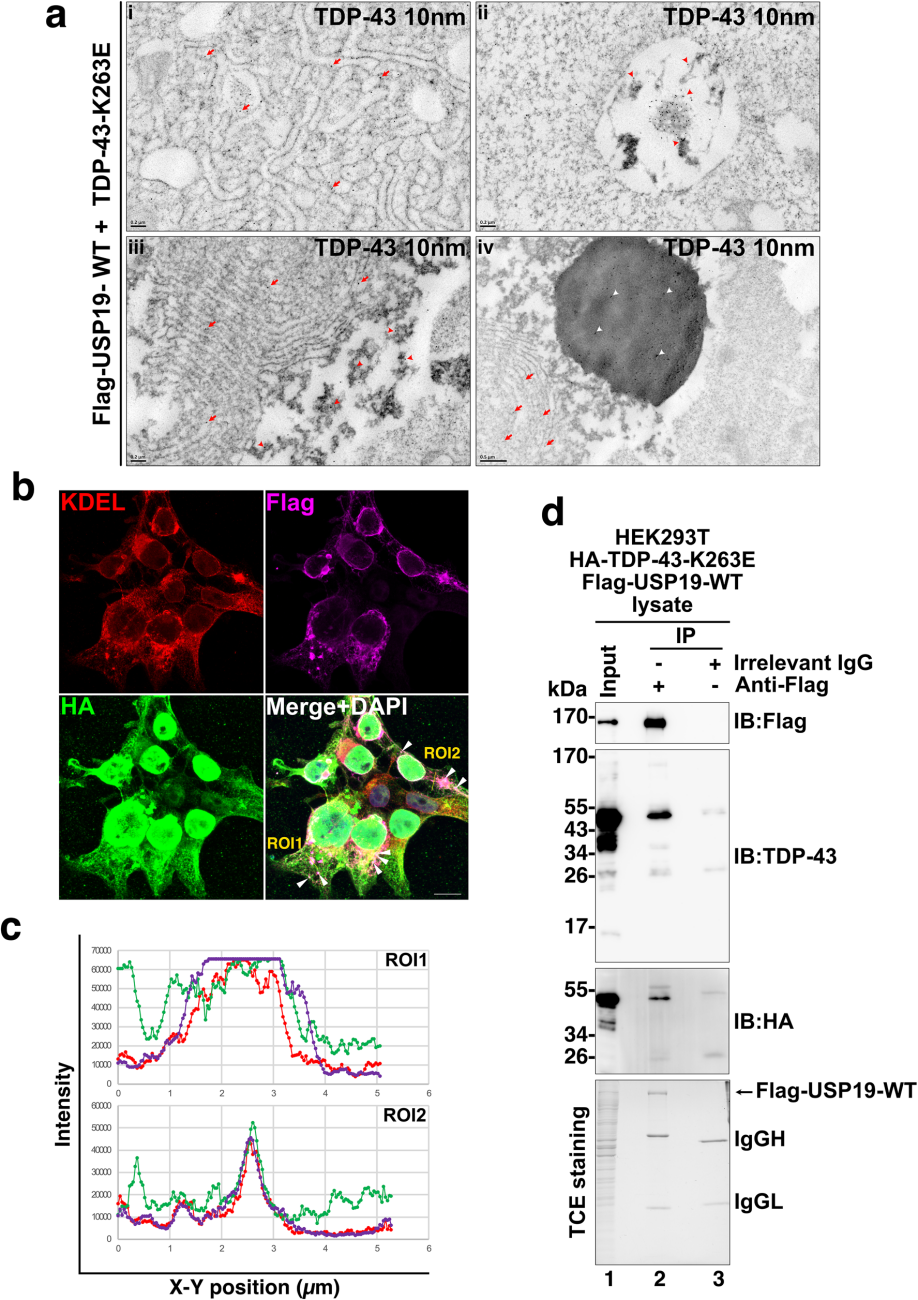
TDP-43-K263E colocalizes with USP19-WT and the KDEL-ER marker and coimmunoprecipitates with USP19-WT.** a** TDP-43 IEM of HEK293T TDP-43-K263E + Flag-WT-USP19 co-expressing cells. Red arrows indicate the TDP-43 gold particles in close contacts with dilated ER structures in the cytoplasm (panel i) or inside autophagic/endosomal compartments (panels iii and iv). Red arrowheads correspond to free TDP-43 aggregates in intracellular compartments (panel ii). White arrowheads correspond to TDP-43 labelling embedded in amorphous structures present in intracellular autophagic/endosomal compartments (panel iv). **b** Confocal immunofluorescence imaging of TDP-43-K263E + Flag-USP19-WT co-expressing cells. Co-expressing cells were labelled with antibodies directed against the ER KDEL marker (red), the Flag-USP19-WT (magenta), the HA for TDP-43-K263E (green) and the DAPI (blue) for nuclear staining. Scale bar is 10 μm. **c** ROI 1–2 are depicted in the merge panel. The plot profiles of the ER-KDEL marker (red) with the Flag-USP19-WT (magenta) and the HA-TDP-43-K263E (green) colocalizations (ROI1 and 2) along the ROI lines were constructed and analyzed using Image J software. White arrowheads show other colocalized signals **d** TDP-43 coimmunoprecipitates with Flag-USP19-WT. Left panel: co-immunoprecipitation experiment was realized on cell lysates from TDP-43-K263E and Flag-USP19-WT co-expressing cells using mouse antibodies directed against the Flag epitope or an irrelevant SARS-CoV2 IgG antibody as negative control. Right panel: Western blotting of HA-TDP-43-K263E and Flag-USP19-WT co-expressing cell lysates (input) using antibodies directed against Flag (for USP19), HA (for TDP-43) and GAPDH

We further investigated the distribution of TDP-43 in USP19-WT co-expressing cells using IEM. The data presented in Fig. 5a showed TDP-43 labeling near the accumulated ER (red arrows in panel i), within the lumen of intracellular compartments most likely corresponding to the aggregates observed in our fractionation gradients (red arrowheads in panels ii and iii), and in close contact with engulfed ER structures (red arrows in panels iii, iv) or trapped into electron dense amorphous structures (white arrowheads in panel iv).

IEM experiments on TDP-43-K263E and USP19-ΔTM co-expressing cells revealed TDP-43 labeling primarily associated with the CEDS and less outside these structures (red arrows in Fig. S8), thus suggesting that part of TDP-43 could be trapped within big CEDS.

Confocal immunofluorescence imaging of cells co-expressing HA-TDP-43-K263E and Flag-USP19-WT confirmed the colocalization of Flag-USP19-WT with both the ER-KDEL marker and HA-TDP-43-K263E (Fig. 5b, c).

To investigate potential physical interactions between Flag-USP19-WT and HA-TDP-43-K263E, anti-Flag immunoprecipitations experiments were conducted on lysates from cells co-expressing Flag-USP19-WT and HA-TDP-43-K263E. The data presented in Fig. 5d show that Flag-USP19-WT coimmunoprecipitates with HA-TDP-43-K263E, while no interaction was observed with a control antibody.

Overall, the physical interaction between USP19 and mutant TDP-43, along with their localization in various intracellular compartments including LC3 positive compartments, further supports the direct involvement of USP19 in the cellular handling and potential secretion of misfolded-TDP-43 species.

### Role of Early autophagic and late endosomal compartments in the TDP-43-K263E secretion mediated by USP19

To better characterize the cellular mechanisms by which TDP-43-K263E is secreted upon USP19-WT expression, the release of TDP-43 was explored through kinetic studies to pinpoint the onset of misfolded TDP-43 secretion. For this purpose, HEK293T cells were co-transfected with constructs as described above, and the secretion of misfolded TDP-43 was monitored by FTA ranging from 24 to 40 h post-transfection. This approach allowed us to identify the initial time point at which TDP-43 secretion becomes detectable, providing valuable insights into the temporal dynamics of this process. The data presented in Fig. S9 revealed that misfolded TDP-43 secretion becomes significant 27 h after transfection and gradually increases, reaching its peak of release at 40 h. Western blotting analyses of corresponding cell lysates revealed an increase of LC3-II protein levels compared to control (co-expression of TDP-43-K263E with USP19–ΔTM), correlating with our EM and confocal immunofluorescence data as well as with previously findings indicating that USP19 is a positive regulator of autophagy [44].

**Fig. 6:**
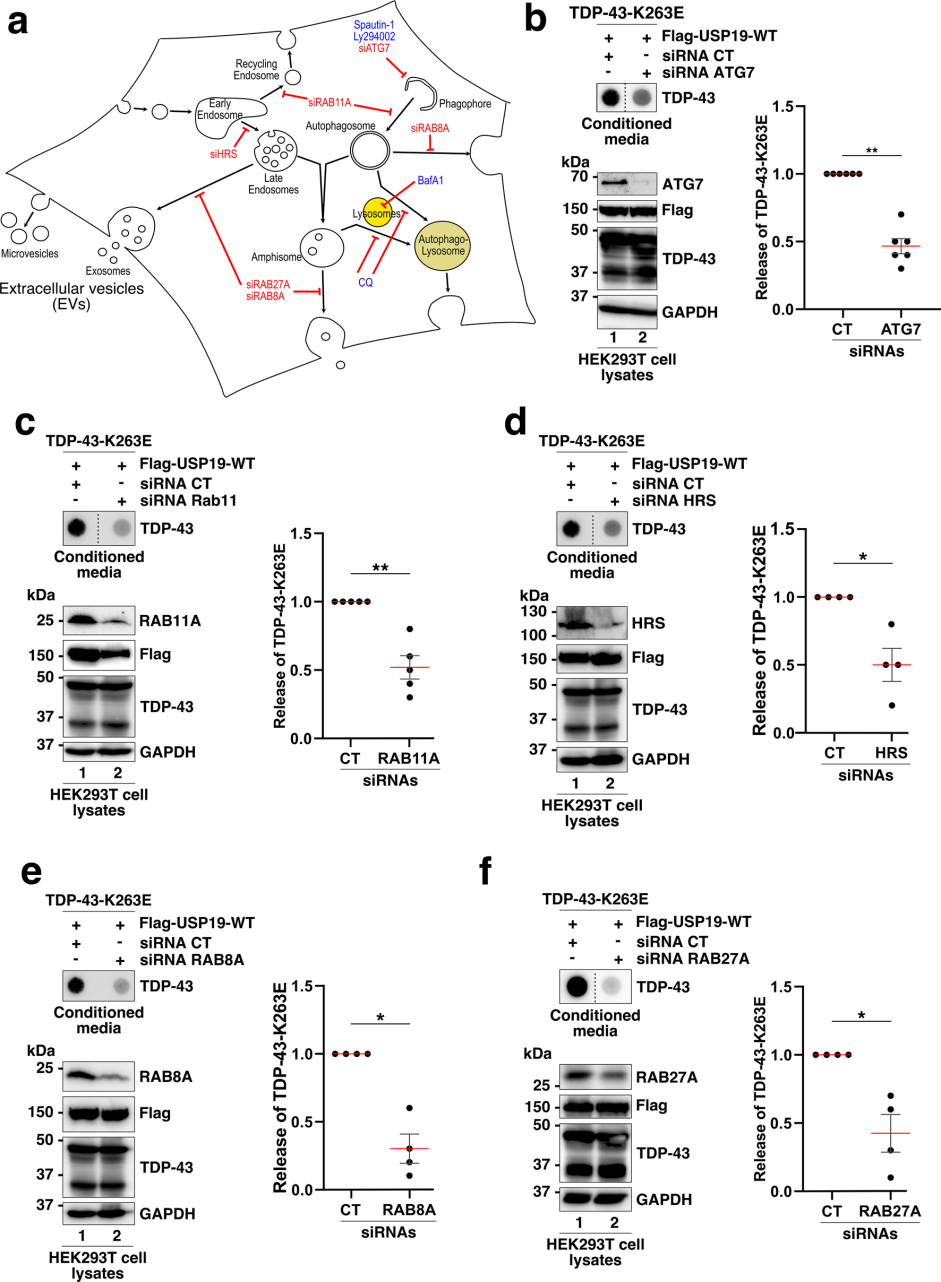
Early autophagic and endosomal compartments are involved in the TDP-43-K263E secretion mediated by USP19-WT.** a** Schematic representation of cellular trafficking and inhibition strategies (pharmacological in blue and siRNAs in red) used in this study to identify potential pathways involved in the TDP-43-K263E secretion mediated by USP19. **b-f** Evaluation of the TDP-43-K263E secretion mediated by USP19 by FTA in presence of siRNAs control (CT) or siRNAs directed against *ATG7*, *RAB11A*, *HRS/HGS*, *RAB8A* or *RAB27A*. Upper panel (secretion/conditioned media): nitrocellulose membranes were probed with an antibody directed against TDP-43. Lower panel (cell expression): Western blotting of cell lysates from siRNAs CT/targets-treated co-expressing cells using antibodies directed against ATG7, RAB11A, HRS/HGS, RAB8, RAB27A or Flag (for USP19), TDP-43, and GAPDH for loading control. Quantification of secreted TDP-43-K263E mediated by USP19-WT in presence of siRNAs CT and targets *ATG7*, *RAB11A*, *HRS/HGS*, *RAB8A* and *RAB27A* are depicted in the right panels of each condition. Data represent mean ± SEM, *n* = 4 to 6 experiments. Significance was assessed by a Mann–Whitney U test (*p < 0.05)

To explore the implication of the autophagic pathway in USP19-mediated secretion of misfolded TDP-43, we examined the effects of pharmacological inhibitors and small interfering RNAs (siRNAs) targeting host factors involved in intracellular trafficking and/or the biogenesis and fate of autophagic compartments (Fig. 6a).

The VPS34 protein (class III phosphatidylinositol 3 kinase PI3K) plays a crucial role in the early steps of autophagosome biogenesis, and its inactivation impairs autophagosome formation[45, 46]. Pharmacological inhibitor of VPS34, Spautin-1 and LY294002, significantly reduced the secretion of misfolded TDP-43 compared to the DMSO control (Fig. S10a, b). Inhibition of VPS34 was confirmed by Western blotting showing the inhibition of S6 phosphorylation, a downstream effector of VPS34.

To confirm the implication of autophagosomes, ATG7, a key factor involved in early autophagosome biogenesis, was silenced using ATG7 siRNAs. The data presented in Fig. 6b revealed that ATG7 siRNA reduced the secretion of aggregated TDP-43. Similarly, RAB11A has also been found to be required for the recruitment and assembly of the autophagic machinery [47]. Consistently, when we conducted a RAB11A siRNA, we observed a significant reduction of USP19-mediated TDP-43-K263E secretion (Fig. 6c).

Altogether, these findings indicate that autophagosomes are involved in USP19-mediated secretion of misfolded TDP-43.

Autophagosomes can fuse with late endosomal compartments to form hybrid amphisomal compartments, and the endosomal sorting complex required for transport ESCRT-0 HRS/HGS component plays a key role in late endosomes and amphisomes biogenesis [48, 49]. We found that silencing HRS/HGS expression efficiently decreased USP19-mediated TDP-43-K263E secretion indicating the involvement of the late endosomal/amphisome compartments (Fig. 6d).

Autophagosomes or amphisomes can fuse with lysosomes to create hybrid structures called autophagolysosomes, where protein enzymatic degradation occurs. To determine if lysosomes play a role in this secretion process, lysosomal enzymatic activity or the capacity of lysosomes to fuse with autophagosomes and amphisomal compartments were inhibited with Bafilomycin A1 (BafA1) or Chloroquine (CQ) respectively. The data presented in Fig. S10a, b showed that USP19-mediated TDP-43-K263E aggregates release was not impaired and even slightly increased compared to the control, suggesting that lysosome-dependent degradation or fusion is not required.

Alternatively, autophagosomes and amphisomes can fuse with the plasma membrane to release their content into the extracellular space. The GTPases RAB8A or RAB27A are key factors in the transport of MVB/late endosomes to the plasma membrane and autophagosome/amphisome mediated secretory autophagy [50, 51]. To determine if these factors modulate the secretion process, RAB8A or RAB27A were silenced using specific siRNAs. The data presented in Fig. 6e, f revealed that silencing of RAB8A and RAB27A significantly reduced USP19-mediated TDP-43-K263E secretion thus confirming their key roles in the late steps of this secretion process.

Altogether, these findings indicate that early autophagic and late endosomal/amphisomal compartments as well as autophagome/amphisome-mediated secretion are involved in the USP19-mediated TDP-43-K263E secretion.

### The vesicular SNARE VAMP7 regulates the secretion of misfolded TDP-43 mediated by USP19

The fusion of intracellular compartments such as late endosomes, amphisomes or autophagosomes with the plasma membrane requires the activity of v- and t-SNARES. These proteins are distributed at the surface of the donor and acceptor intracellular compartments and the cytoplasmic leaflet of the plasma membrane, respectively. In the context of exocytosis [52–55], vesicle-associated membrane protein 7 (VAMP7) is a v-SNARE associated with late endosomes, lysosomes and RAB11A positive compartments. VAMP7 interacts with both RAB8 and RAB11A [56]. Recent studies have also shown that VAMP7 is involved in secretory reticulophagy/ER-phagy [57], where elements of the ER such as reticulons, can be released in a VAMP7-dependent manner [58]. VAMP7 protein has also been implicated in autophagosome biogenesis [59]. These data prompted us to investigate the role of VAMP7 in the USP19-mediated TDP-43-K263E secretion.

**Fig. 7:**
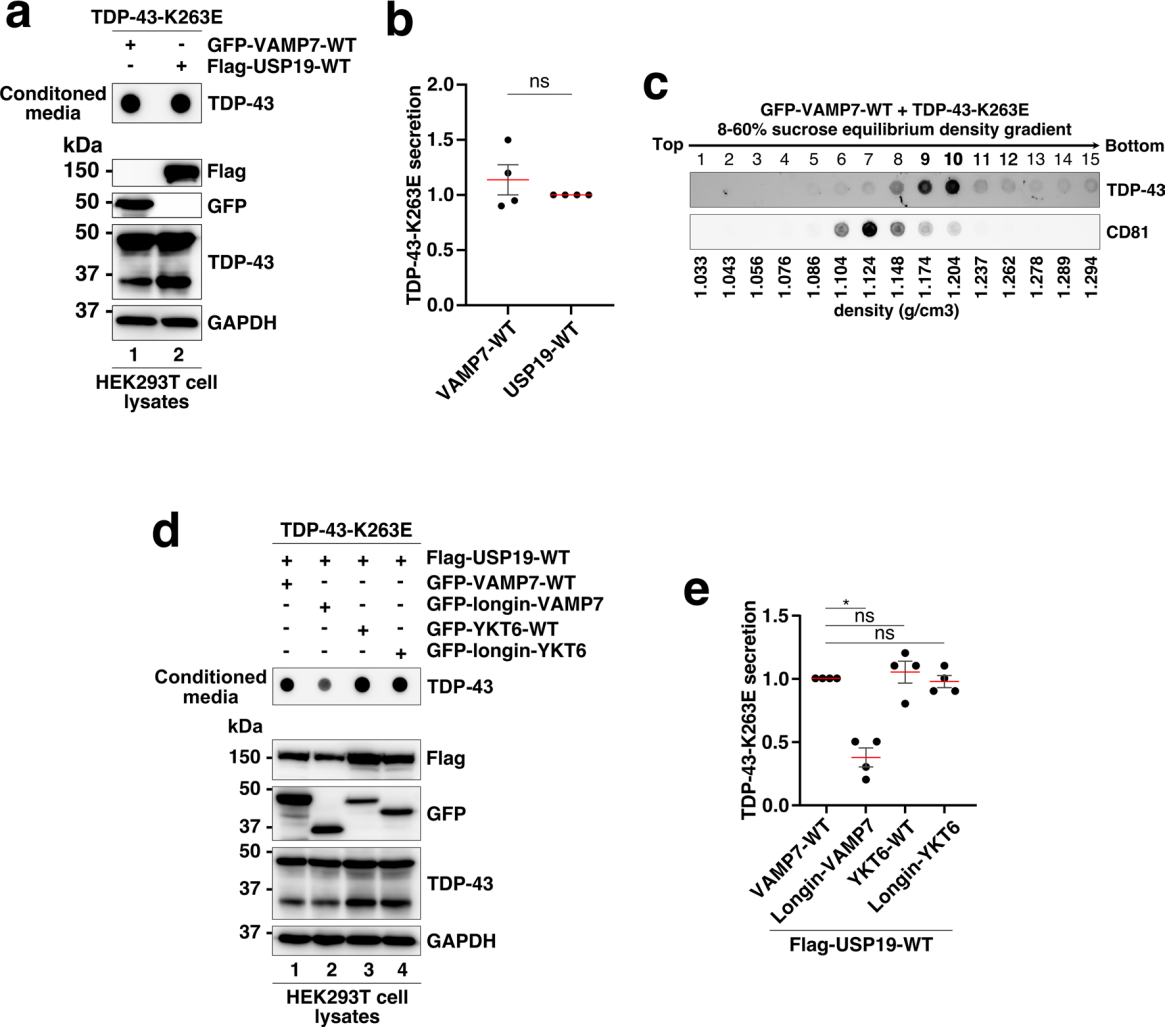
VAMP7 is a modulator of the TDP-43-K263E secretion mediated by USP19-WT. **a** Evaluation of the TDP-43-K263E secretion mediated by EGFP-VAMP7-WT and Flag-USP19-WT by FTA. Upper panel (secretion): nitrocellulose membranes were probed with an antibody directed against TDP-43. Lower panel (cell expression): Western blotting of cell lysates from co-expressing cells using antibodies directed against Flag (for USP19), TDP-43, GFP (for VAMP7-WT) and GAPDH for loading control. **b** Quantification of secreted TDP-43-K263E in presence of GFP-VAMP7-WT or Flag-USP19-WT expression. Data represent mean ± SEM, *n* = 4 experiments. Significance was assessed by a Mann–Whitney U test, ns = not significant. **c** Sucrose equilibrium density gradient fractionation of conditioned medium. The 120,000xg pellet (p120K) from conditioned media of TDP-43-K263E + GFP-VAMP7-WT co-expressing cells were fractionated through a 8–60% linear sucrose equilibrium density gradient and fractions (× 15), recovered from the top were analyzed by Western blotting using anti-TDP-43 or anti-CD81 antibody. Fraction density values (g/cm^3^) are depicted at the bottom panel. **d** Evaluation of TDP-43-K263E secretion mediated by USP19-WT in presence of EGFP- VAMP7-WT, EGFP-Longin-VAMP7, EGFP-YKT6-WT or EGFP-Longin-YKT6 by FTA. Upper panel (secretion/conditioned medium): nitrocellulose membranes were probed with an antibody directed against TDP-43. Lower panel (cell expression): Western blotting of cell lysates from co-expressing cells using antibodies directed against Flag (for USP19), TDP-43, GFP (for VAMP7s and YKT6s) and GAPDH for loading control. **e** Quantification of secreted TDP-43-K263E mediated by Flag- USP19-WT in presence of VAMP7 or YKT6-WT and -Longins expression. Data represent mean ± SEM, *n* = 4 experiments. Significance was assessed by a Mann–Whitney U test (*p < 0.05), ns = not significant

We first examined the impact of GFP-VAMP7-WT overexpression on the secretion of misfolded TDP-43-K263E. The data presented in Fig. 7a, b showed that overexpression of VAMP7-WT significantly enhances the secretion of TDP-43-K263E, mimicking the effect of USP19-WT and correlating with previously published data [26]. When conditioned media from GFP-VAMP7-WT and TDP-43-K263E co-expressing cells were fractionated through an 8–60% sucrose equilibrium density gradient (Fig. 7c), TDP-43 mainly localized in fractions of high density (9 and 10), similar to what was observed for USP19, compared to fractions of lower density (fractions 6 to 8) that contained the CD81 positive EVs marker. This suggests that VAMP7-WT mediates TDP-43-K263E secretion in a manner similar to USP19-WT.

VAMP7 possesses a long N-terminal extension of 90 residues called the Longin domain, responsible of the auto-inhibitory regulation of the v-SNARE, downregulating the formation of the SNARE complex [54, 60]. Consequently, overexpression of the Longin domain impairs the fusion mechanism between VAMP7-positive compartments and the plasma membrane [61, 62].

We took advantage of this feature to investigate the role of VAMP7 in USP19-mediated TDP-43-K263E secretion. For this purpose, we characterized cells expressing TDP-43-K263E, USP19-WT and either GFP-VAMP7-WT or GFP-VAMP7-Longin and the conditioned media were analyzed by FTA. The data presented in Fig. 7d, e showed that overexpression of the GFP-VAMP7-Longin significantly reduces the USP19-mediated misfolded TDP-43-K263E secretion compared to the control. To validate the specific impact of the VAMP7 Longin, similar experiments were conducted using YKT6, another v-SNARE containing a Longin domain [54, 60] involved in the fusion of autophagosomes with the lysosomes [37, 63, 64]. Overexpression of GFP-YKT6-WT or GFP-YKT6-Longin did not impair the USP19-mediated TDP-43-K263E secretion, indicating a specific impact of VAMP7 in USP19-mediated secretion (Fig. 7d, e). This last set of data confirm, once more, that the final intervention of lysosomes is not essential for the USP19-mediated secretion of misfolded TDP-43 and that VAMP7 regulates the secretion of misfolded TDP-43 mediated by USP19.

As previously depicted, VAMP7 appears to play a key role in the autophagosome biogenesis [59] as well as during the late steps of the fusion process between the intracellular compartments and the plasma membrane [65]. Since VAMP7 alone, promotes, like USP19, the secretion of misfolded TDP-43, we compared whether combined expression of USP19-WT and VAMP7-WT could display a synergistic impact on the secretion of misfolded TDP-43. For this purpose, conditioned media from HEK293T cells co-expressing the mutant GFP-TDP-43-ΔNLS-Δ187-192 with either Flag-USP19-WT alone or RFP-VAMP7-WT alone or combined (USP19-WT + VAMP7-WT) were analyzed by FTA. The data presented in Fig. S11a-c failed to identify a significant difference between the different secretion conditions thus indicating that no additive effect of VAMP7-WT was observed, and suggesting that VAMP7 did not act in a parallel pathway of USP19.

### The DNAJC5/CSPα chaperone protein modulates the release of TDP-43 aggregates mediated by USP19

**Fig. 8:**
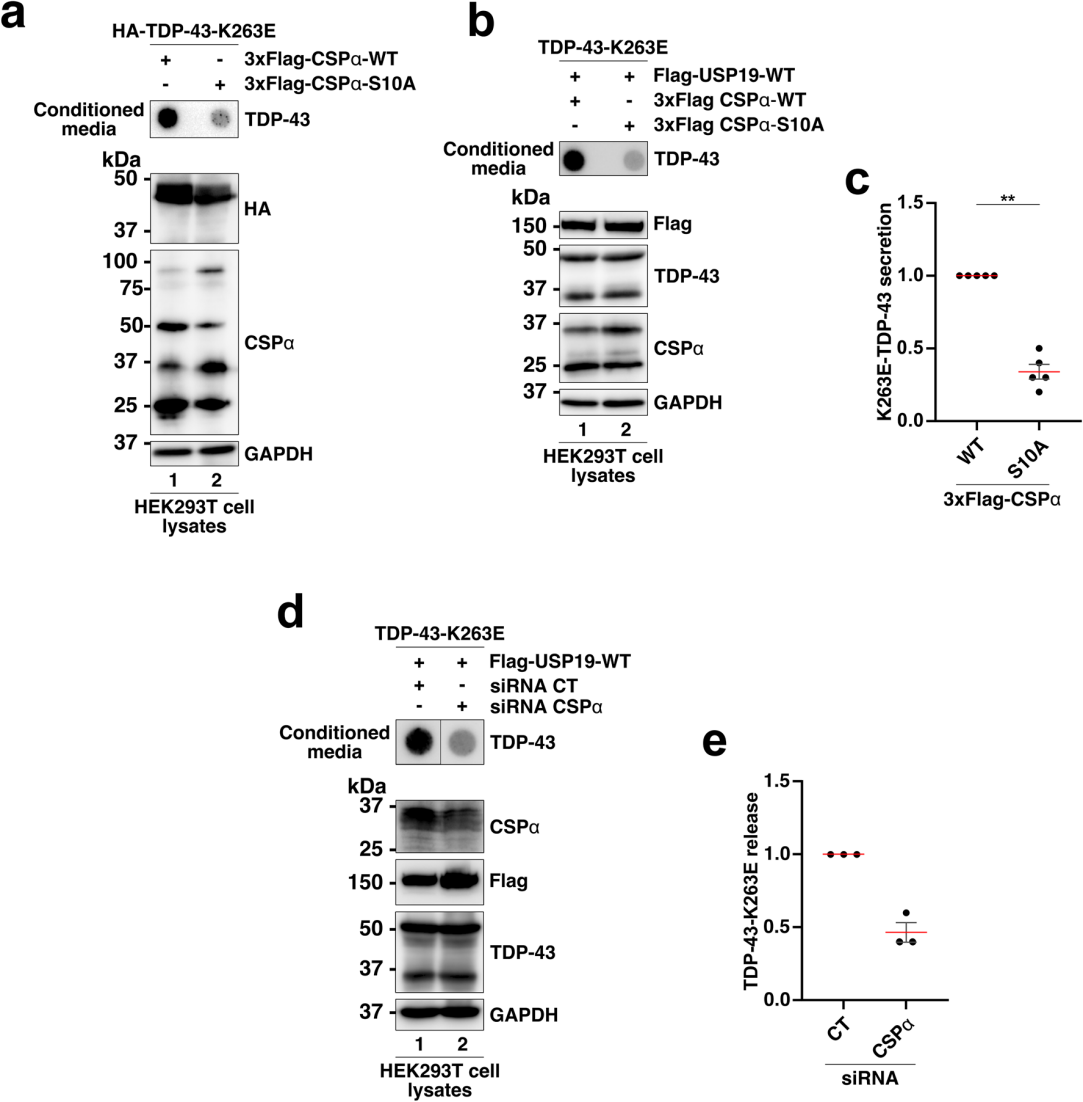
DNAJC5/CSPα is a modulator of the misfolded TDP-43-K263E secretion mediated by Flag-USP19-WT. **a** Evaluation of TDP-43-K263E secretion mediated by 3x-Flag-CSPα-WT or the phospho-deficient CSPα-S10A by FTA. Upper panel (secretion/conditioned media): nitrocellulose membranes were probed with an antibody directed against TDP-43. Lower panel (cell expression): Western blotting of cell lysates from co-expressing cells using antibodies directed against HA (for HA tagged TDP-43), CSPα and GAPDH for loading control. **b** Evaluation of the TDP-43-K263E secretion mediated by USP19-WT in presence of CSPα-WT or CSPα-S10A mutant by FTA. Upper panel (secretion/conditioned media): nitrocellulose membranes were probed with an antibody directed against TDP-43. Lower panel (cell expression): Western blotting of cell lysates from co-expressing cells using antibodies directed against Flag (for Flag-USP19-WT), TDP-43, CSPα and GAPDH for loading control. **c** Quantification of secreted TDP-43-K263E mediated by USP19-WT in presence of CSPα-WT or the phosphor-deficient CSPα-S10A expression. Data represent mean ± SEM, *n* = 5 experiments. Significance was assessed by a Mann–Whitney U test (**p < 0.001). **d** Evaluation of TDP-43-K263E secretion mediated by Flag-USP19-WT in presence of siRNAs CT or siRNAs directed against *CSPα* by FTA. Upper panel (secretion/conditioned media): nitrocellulose membranes were probed with an antibody directed against TDP-43. Lower panel (cell expression): Western blotting of cell lysates from co-expressing cells treated with siRNAs CT or against CSPα using antibodies directed against CSPα, Flag (for Flag-USP19-WT), TDP-43, or GAPDH for loading control. **e** Quantification of secreted TDP-43-K263E mediated by USP19-WT in presence of siRNAs CT or siRNAs CSPα. Data represent mean ± SEM, *n* = 3 experiments

The DNAJC5/CSPα chaperone protein regulates the secretion of neurodegenerative-associated proteins including α-synuclein, the microtubule-associated protein tau and the pathological A315T or the Q343R TDP-43 variants [66]. In addition, CSPα was previously found to chaperone MAPS client proteins to the extracellular space [28]. We first confirmed that CSPα-WT overexpression was sufficient to promote the secretion of misfolded TDP-43-K263E in absence of USP19 overexpression, whereas its phospho-deficient CSPα-S10A mutant did not (Fig. 8a), as previously observed for the tau protein [66]. Based on these findings, we next investigated the impact of CSPα-WT and -S10A on USP19-mediated TDP-43-K263E secretion. The data presented in Fig. 8b, c revealed that the phospho-deficient S10A mutant impairs the secretion of misfolded TDP-43.

To validate the role of CSPα as a modulator of USP19-mediated TDP-43-K263E secretion, siRNAs were used to silence endogenous CSPα expression. Analysis of TDP-43 secretion by FTA showed that CSPα silencing significantly decreased TDP-43-K263E secretion, thus confirming that CSPα can modulate the MAPS process, and, in this context, the USP19-mediated secretion of misfolded TDP-43 (Fig. 8d, e).

## Discussion

Here, we investigated the impact of the ER-USP19 on the management of misfolded TDP-43, the major component of cytoplasmic inclusions found in neurons and glial cells in ALS and ALS-FTLD. Using two different cell models (HEK293T and human neuroblastoma SH-SY5Y cell lines), we found that USP19 overexpression significantly enhances the secretion of TDP-43 mutants (the familial ALS TDP-43-K263E variant and the truncated TDP-43–ΔNLSΔ187-192- mutant) as free aggregates or soluble misfolded TDP-43. This process requires both the ER-membrane anchoring and the ubiquitin peptidase activity of USP19. These findings align with previous studies suggesting that ER-associated USP19 plays a crucial role in regulating protein aggregation, and the secretion of proteins associated with neurodegenerative disorders including α-synuclein [26, 28, 31].

USP19 is the only member of the USP family anchored to the cytosolic face of the ER membrane [26, 27]. A second USP19 isoform (USP19_b 1–1281) lacking an ER anchoring transmembrane domain was shown to promote the aggregation of polyglutamine-expanded Ataxin3 (Atx3) and Huntingtin [67]. Since our results show the requirement of the ER anchoring transmembrane domain of USP19, this second isoform is unlikely to play a role in the secretion of misfolded TDP-43. Strikingly, overexpression of the non-anchored USP19–ΔTM mutant, induces the formation of large CEDS containing trapped TDP-43. It will be interesting in the future to understand how this cytosolic USP19 mutant promotes the formation of these giant aggregated structures and to characterize their exact composition.

USP19 overexpression did not promote the secretion of the aggregation prone GFP-TDP-43^219–414^-CTF mutant, while it efficiently facilitated the secretion of GFP-TDP-43-ΔNLSΔ187-192. These findings are consistent with the observations by Xu et al*.,* (2018) showing that GFP-Q103 polyglutamine is not secreted compared to the GFP-Q25 [28]. It suggests that USP19-dependent secretion may preferentially export soluble misfolded proteins or smaller protein aggregates [28]. The reason why larger aggregates are not managed by USP19 remains unclear. One hypothesis is that ubiquitin sequestered within dense and more stable inclusions induced by GFP-TDP-43^219–414^-CTF may be less accessible to deubiquitylation by USP19. In contrast, smaller inclusions and soluble oligomers would be deubiquitinated by USP19 and subsequently processed through the MAPS pathway. Consistently, we found that USP19-WT promotes the secretion of free aggregates as well as soluble misfolded TDP-43 in the extracellular medium. Alternatively, since canonical ubiquitination takes place on lysine residues, the lack of secretion of the GFP-TDP-43^219–414^-CTF mutant in presence of USP19-WT expression, may also be attributed to the fact that this truncated form of TDP-43 lost most of its lysine residues. Indeed, most of ubiquitination sites on TDP-43 have been identified within or near the RRM domains and 16 over 20 total lysine residues were distributed between the N-terminal residues 1 to 218 [41]. Connected to this point, we confirmed that TDP-43-K263E is ubiquitinated when the ubiquitin peptidase deficient mutant was expressed whereas, in presence of USP19-WT expression, deubiquitination and secretion of TDP-43-K263E was observed. These findings can correlate with the deficient secretion observed for the TDP-43-CTF fragment.

Many studies have demonstrated the secretion of TDP-43 in association with extracellular vesicles, particularly the smaller ones generated in late endosomes, so-called exosomes [13, 19, 22–24]. In contrast, TDP-43 is released as free aggregates upon cell death in neuronal cultures [25]. Our investigations revealed that USP19-WT promotes the secretion of soluble and aggregated forms of misfolded TDP-43 without associated cell mortality. While a faint TDP-43 signal was detected in CD81/CD63 exosomal positive fractions, most of the secreted misfolded TDP-43 was concentrated in fractions of higher density. This indicates that USP19 does not promote the release of misfolded TDP-43 through the exosomal pathway. These findings strongly suggest that the USP19-mediated MAPS pathway conducts a specific sorting of misfolded TDP-43, leading to its secretion as soluble and free aggregates rather than through exosomes.

The exact molecular and cellular mechanisms by which cells select misfolded proteins for secretion via the USP19 dependent MAPS pathway remain enigmatic. It was proposed that USP19, anchored at the cytosolic side of the ER membrane, deubiquitinates misfolded proteins and, in conjunction with DNAJC5/CSPα and HSC70 chaperones, redirect them to the secretory late endosomal pathway [26, 28]. The fact that TDP-43 was found to be released in a VAMP7-dependent manner is certainly coherent with this notion.

In our study, we observed that inhibiting DNAJC5/CSPα function using a dominant negative construct (S10A-CSPα), or through DNAJC5/CSPα siRNAs, significantly reduced the release of misfolded TDP-43 mediated by USP19. This confirms that DNAJC5/CSPα is a key modulator of the USP19 associated MAPS pathway and can promote the secretion of misfolded TDP-43, both in the presence or absence of USP19 overexpression.

TEM investigations revealed a significant accumulation of dilated ER structures in HEK293T cells co-expressing USP19-WT and TDP-43-K263E. Our RNA-seq investigations confirmed alterations linked to the ER organelle in the context of USP19 overexpression. These findings align well with previously published data indicating the close relationship between USP19 expression and the ER stress [27].

Consistently, the various experimental approaches (TEM, IEM and confocal microscopy) we used, visualized TDP-43 in close contact with ER membrane as well as LC3 positive structures accumulated into secretory intracellular compartments. Additionally, co-immunoprecipitation experiments demonstrated interactions between USP19 and TDP-43 thus suggesting that part of TDP-43 could be secreted in a form of a tri-complex ER-USP19-TDP-43 engulfed into secretory compartments.

To gain a clearer understanding of the cellular mechanisms involved in the misfolded TDP-43 secretion via the MAPS pathway, we evaluated the impact of the Spautin1 and LY294002 inhibitors on the early steps of phagophores/autophagosomes biogenesis [45, 46]. Our results showed a strong inhibition of USP19-mediated misfolded TDP-43 secretion, indicating a crucial role of early autophagosomal compartments. However, it is important to note that previous studies have shown that Spautin1 and LY294002 can also impair various members of the USP family such as USP10, USP13 or USP18 [68, 69]. Therefore, we cannot rule out the possibility that these inhibitors might also inhibit USP19. Nevertheless, the implication of early autophagy processes was further supported by a significant decrease in USP19-mediated misfolded TDP-43 secretion upon inhibition of ATG7, RAB11A or VAMP7 components, all of which are involved in the early steps of phagophore/autophagosome biogenesis [47, 59, 70]. These findings were also corroborated by an increase of lipidated LC3 observed in our kinetic studies and correlated with previously published data [44, 71], as well as the fact that USP19 promotes the autophagic flux [44].

Autophagosomes compartments can fuse with late endosomes and/or multivesicular bodies to form hybrid compartments known as amphisomes. Lee et al*.,* (2016) identified Rab9 late endosomes as a key compartment involved in the MAPS unconventional secretion pathway. The ESCRT-0 component HRS/HGS, is crucial for amphisome biogenesis [48]. Here, we found that silencing of ATG7 or HRS/HGS significantly impair secretion of misfolded TDP-43 upon USP19 expression, thus underscoring the importance of both compartments and not only late endosomes.

Autophagosomes and late endosomes/amphisomes are essential intracellular compartments involved in secretory autophagy, and their transport to, or their fusion with, the plasma membrane is regulated by the RabGTPases RAB8A and RAB27A and the v-SNARE protein VAMP7 [50, 51, 54, 61, 72]. Silencing RAB8A or RAB27A, or inhibiting VAMP7-dependent exocytosis using the VAMP7-Longin domain, significantly reduced USP19-mediated secretion of misfolded TDP-43. Accordingly, a recent study revealed that VAMP7 plays a pivotal role in the secretory ER-phagy by facilitating the exocytosis of ER-cargos containing LC3 interacting region (LIR-domain) such as Reticulons and Atlastin through late endosomes/amphisomes [58]. The role of DNAJC5/CSPα in this process has not been investigated, but it is conceivable that cargos could be shuttled into the lumen of late endosomes and or amphisomes with the assistance of this cochaperone as previously shown for α-synuclein [73].

Accordingly, the release of α-synuclein multimers not associated with EV was found to be also VAMP7-dependent using Longin expression [74].

Lysosomes play a crucial role in degradative and secretory autophagy processes. Our data suggest that lysosomes are not key compartments involved in the USP19-mediated secretion of misfolded TDP-43, which aligns with previously published findings [26]. The moderate increase in TDP-43 secretion that was observed in presence of Baf A1 and CQ inhibitors, can be attributed to the fact that inhibition of the lysosomal ATPase is known to induce lysosome fusion to the cell surface and secretion of lysosomal content, including both soluble and aggregated proteins such as α-synuclein [73, 74].

Overall, our findings indicate that misfolded TDP-43 secretion is managed by the secretory autophagy/ER-phagy via the engulfment of ER-USP19-misfolded TDP-43 complexes by phagophores, or through late endosomes or alternatively through the hybrid amphisomes compartments. This secretion process would be modulated by additional factors such as RAB11A, RAB8A, RAB27A and VAMP7 in a similar way to what was described for Annexin A2 [75].

ER-phagy can be mediated through the implication of different receptors anchored to the cytosolic face of the ER membrane and with the capacity to interact with LC3 (through the LIR domain) or SQSTM1/p62 [76]. We did not observed upregulation of these receptors at the mRNA level upon of USP19 overexpression thus suggesting that these receptors are not induced in our context. USP19 displays similarities with these receptors since it is anchored to the ER. In addition, manual searching for canonical LIR consensus amino acid sequence (F or W or Y)-x-x-(L or I or V) (where F, W, Y, L, I or V are phenylalanine, tryptophan, tyrosine, leucine, isoleucine or valine respectively and x any amino acid residues) [77] revealed the presence of potential LIR domains in USP19 (P.Leblanc personal communication). In this context, we cannot exclude that the ER-associated USP19 itself, could act as an ER-phagy receptor. In this process, ER-tubules and Reticulons in particular could play an important role as previously suggested [78] and this will require further investigation.

To conclude, we acknowledge that this study displays some limitations. One weakness of our approach is the immortalized cell lines used in our study that not fully represent the physiological conditions of neurons in the central nervous system. Importantly, our study is the first to investigate USP19's impact on pathological or mutant TDP-43. Our study was built upon previous research on USP19 and the MAPS secretion pathway on misfolded-GFP or α-synuclein, which primarily utilized HEK293T cells. We extended this work by validating USP19-mediated secretion of misfolded TDP-43 in the human SH-SY5Y neuroblastoma cell line. Our main goal was first to determine if USP19 could mediate the secretion of misfolded TDP-43 through the MAPS pathway and second to mechanistically understand the cellular and molecular process by which USP19 promotes this secretion.

Despite their limitations, both of the cellular models used in this study offer significant advantages over more complex systems like motor neurons derived from induced pluripotent stem cells (iPSCs) which are difficult to transfect and often fail to produce significant amounts of aggregated TDP-43 even when derived from ALS patients.

Interestingly, the HEK293 cellular model exhibit neuronal lineage characteristics, expressing neurofilament and various neuronal proteins. Their ability to propagate neurotropic viruses, induce synaptogenesis and exhibit functionality in neuron-specific voltage-gated channels supports their neuronal lineage phenotype [79, 80]. Previous published studies, including our, demonstrated that HEK293T or SH-SY5Y cell lines can mirror co-aggregation of mutant Huntingtin and TDP-43 in disease patient brains [81, 82]. This finding underscores the value of HEK293T cells in mimicking alterations seen in patient brains and their utility as an effective tool for elucidating mechanisms underlying protein aggregation and secretion with prion-like proteins. In conclusion, while we recognize the limitations of our cellular models, their strengths in terms of manipulability, protein expression, and ability to recapitulate certain disease-relevant phenotypes make them valuable tools for our study.

Overall, this study describes a new pathway of misfolded TDP-43 secretion. It will be of interest to determine if TDP-43 secreted via this pathway can be transmitted to neighboring cells and therefore participate to the spreading of the pathology in vivo. In this way, USP19 appears to be an interesting candidate that needs, in the future, to be investigated in vivo in ALS mouse models as well as in ALS and ALS-FTLD patient brains.

## Supplementary Information

Below is the link to the electronic supplementary material.

Supplementary file1 (DOCX 12222 kb)

Supplementary file2 (XLSX 1807 kb)

## Data Availability

Materials described in the manuscript, including all relevant raw data, will be freely available to any researcher. The datasets generated during and/or analyzed during the current study are available from the corresponding author on reasonable request.

## Abbreviations

ALS: Amyotrophic lateral sclerosis
Baf A1: Bafilomycin A1
CEDS: Compact electron dense structures
CQ: Chloroquine
DMSO: Dimethyl sulfoxide
ER: Endoplasmic reticulum
ESCRT: Endosomal sorting complex required for transport
EV: Extracellular vesicle
FTA: Filter trap assay
FTLD: Fronto temporal lobar degeneration
GFP: Green fluorescent protein
HGS/HRS: Hepatocyte growth factor-regulated tyrosine kinase substrate
IF: Immunofluorescence
IEM: Immunogold electron microscopy
kDa: Kilo Dalton
MAPS: Misfolded associated protein secretion
NLS: Nuclear localization signal
Sark-sol: Sarksosyl soluble
Sark-ins: Sarkosyl insoluble
TDP-43: Tar DNA binding protein of 43 kDa
TEM: Transmission electron microscopy
USP19: Ubiquitin-specific peptidase 19
VAMP: Vesicle-associated membrane protein
WT: Wild type
