# Supplementary material for: Enhanced secretion of the amyotrophic lateral sclerosis ALS-associated misfolded TDP-43 mediated by the ER-ubiquitin specific peptidase USP19
Source: Cell Mol Life Sci. 2025 Feb 13;82(1):76. doi: 10.1007/s00018-025-05589-w (PMC11825969; doi:10.1007/s00018-025-05589-w)
Supplement: Supplementary file 1 — Supplementary file1 (DOCX 12222 kb) [file 18_2025_5589_MOESM1_ESM.docx]

**Supplementary Fig. S1** Flag-USP19-WT but not Flag-USP19-ΔTM colocalizes with the ER-KDEL marker. **a** HEK293T cells co-expressing TDP-43-K263E and Flag-USP19-WT or -ΔTM or were analyzed by immunofluorescence confocal microscopy using antibodies directed against Flag (red) or the ER-KDEL marker (green). White lines ROI1&2 correspond to region of interest. Scale bar is 10 µm. Nuclei were counterstained with DAPI. **b** ROI 1&2 are depicted in the merge panels. The plot profiles of Flag-USP19-WT and ER-KDEL marker colocalization (ROI1) and Flag-USP19-ΔTM and KDEL absence of colocalization (ROI 2) along the ROI lines were constructed and analyzed using ImageJ software. **c** HEK293T cells co-expressing TDP-43-K263E and Flag-USP19-WT or -ΔTM or were analyzed by immunofluorescence confocal microscopy using antibodies directed against Flag (red) or the GM130 (green) Golgi-marker. White lines ROI1&2 correspond to region of interest. Scale bar is 10 µm. Nuclei were counterstained with DAPI. **d** ROI 1&2 are depicted in the merge panels. The plot profiles of Flag-USP19-WT or -ΔTM and Golgi-GM130 marker absence of colocalizations (ROI1) and (ROI 2) respectively along the ROI lines were constructed and analyzed using ImageJ software.

**Supplementary Fig. S2** The ER-anchored USP19 promotes misfolded HA-TDP-43-K263E secretion in HEK293T cells. **a** Evaluation by FTA of aggregated HA-TDP-43-K263E secretion in presence or absence of Flag-USP19-WT or Flag-USP19-ΔTM expression. Upper panel (secretion/conditioned media): nitrocellulose membranes were probed with an antibody directed against TDP-43 or HA. Lower panel (cell expression): Western blotting of cell lysates from co-expressing cells using antibodies directed against Flag (for USP19), TDP-43, HA (for HA-TDP-43-K263E) and GAPDH for loading control. **b** Quantification of secreted HA-TDP-43-K263E mediated by US1P19. Data represent mean ± SEM, *n* =5 experiments. Significance was assessed by a Mann-Whitney U test (**p < 0.001). **c** Co-expression of Flag-USP19-WT or Flag-USP19-ΔTM with HA-TDP-43-K263E does not affect cell viability. Data represent mean ± SEM, *n* = 3 experiments. **d** Western blotting of CRISPR-Cas9 USP19-KO clones cell lysates. CT is HEK293T control cells; C43, C44 and C66 correspond to independent USP19-KO cell clones. **e** Misfolded HA-TDP-43-K263E is secreted in USP19-KO cells (clone C44) overexpressing Flag-USP19-WT.

**Supplementary Fig. S3** The ER-anchored USP19 promotes the secretion of EGFP-TDP-43-ΔNLSΔ187-192 aggregates but not EGFP-TDP-43-CTF aggregates. **a** Schematic representation of EGFP-TDP-43 mutants used in this study. The EGFP tag is located at the N-terminus of the protein. CTF = C-terminal fragment; LCD = low complexity domain; NLS = nuclear localization sequences; NTD = N-terminal domain; RRM = RNA recognition motif. Deletions of NLS and 187-192 sequence are depicted by red crosses. For the EGFP-TDP-43-CTF there is a deletion of the N-terminus sequence (1-207). HEK293T cells were transfected with EGFP-TDP-43-ΔNLSΔ187-192 or EGFP-TDP-43-CTF constructs and were analyzed by direct fluorescence. Blue signal corresponds to DAPI staining and green signal to EGFP. Scale bar is 10 µm. **b** Filter trap assay of TDP-43 aggregates levels in conditioned media from HEK293T cells overexpressing EGFP-TDP-43-ΔNLSΔ187-192 and the indicated Flag-USP19 or empty vector. Upper panel (secretion): nitrocellulose membranes were probed with an antibody directed against TDP-43. Lower panel (cell expression): Western blotting of cell lysates from co-expressing cells using antibodies directed against Flag (for USP19s), TDP-43, and GAPDH for loading control. **c** Quantification of secreted EGFP-TDP-43-ΔNLSΔ187-192. Data represent mean ± SEM, *n* = 8 experiments. Significance was assessed by a Mann-Whitney U test (***p < 0.0001). **d** Filter trap assay of TDP-43 aggregates levels in conditioned media from HEK293T cells overexpressing EGFP-TDP-CTF and the indicated Flag-USP19s or empty vector. Upper panel (secretion/conditioned media): nitrocellulose membranes were probed with an antibody directed against TDP-43. Lower panel (cell expression): Western blotting of cell lysates from co-expressing cells using antibodies directed against Flag (for USP19s), TDP-43, and GAPDH for loading control. **e** The p120K pellet of conditioned media from cells co-expressing USP19-WT and EGFP-TDP-43-ΔNLSΔ187-192 was fractionated through a 9-60% linear sucrose density gradient. Fractions were analyzed by Western blotting as above using anti-TDP-43, anti-CD81 and anti-CD63 antibodies. Fraction density values (g/cm3) are depicted at the bottom panel. **f** Immunogold electron microscopy of EGFP-TDP-43-ΔNLSΔ187-192 positive fractions. Positive fractions (9-11) containing EGFP-TDP-43-ΔNLSΔ187-192 were pooled and analyzed by IEM using anti-TDP-43 labelled with a secondary antibody coupled with 10 nm gold particle. Scale bars is 100 nm.

**Supplementary Fig. S4** The ER-anchored USP19 promotes the secretion of misfolded TDP-43-K263E in the human neuroblastoma SH-SY5Y cellular model. **a** Evaluation of misfolded TDP-43 secretion by Filter trap assay (FTA). Presence of aggregated TDP-43 in conditioned media from SH-SY5Y cells overexpressing the TDP-43-K263E and the Flag-USP19-WT or the Flag-USP19-ΔTM constructs was monitored by FTA. Upper panel (secretion): nitrocellulose membranes were probed with an antibody directed against TDP-43. Lower panel (cell expression): Western blotting of cell lysates from co-expressing cells using anti-Flag (for USP19), -TDP-43 and -GAPDH antibodies for loading control. **b** Quantification of secreted TDP-43-K263E upon USP19s expression. Data represent mean ± SEM, *n* = 3 experiments. Significance was assessed by a Mann-Whitney U test (**p < 0.001).

**Supplementary Fig. S5** Absence of secretion of TDP-43-K263E in Flag-USP19-ΔTM expression context. Sucrose equilibrium density gradient fractionation of conditioned medium. The 120,000xg pellet (p120K) from conditioned media of TDP-43-K263E and Flag-USP19-ΔTM co-expressing cells were fractionated through a 9-60% linear sucrose equilibrium density gradient and fractions (x15), recovered from the top were analyzed by Western blotting using anti-TDP-43 or anti-CD81 and CD61 antibody. Fraction density values (g/cm3) are depicted at the bottom panel.

**Supplementary Fig. S6** USP19-WT promotes the secretion of soluble misfolded TDP-43 **a** Aggregated TDP-43 levels in conditioned media from HEK293T cells overexpressing HA-TDP-43-K263E with the Flag-USP19-WT or the Flag-USP19-ΔTM were precleared to eliminate cellular debris and ultracentrifuged at 120,000xg pellet (p120K pellet) and analyzed by immunoblotting (lanes 3 and 4) using antibody directed against TDP-43. Cellular lysates (lanes 1 and 2) were analyzed by Western blotting and probed by antibodies directed against HA (for TDP-43), Flag (for USP19) and GAPDH (loading control). **b** FTA of conditioned media from HA-TDP-43-K263E/Flag-USP19-WT or HA-TDP-43-K263E/Flag-USP19-ΔTM before and after ultracentrifugation in absence SDS detergent. Immunoblotting of the membrane using the anti-HA antibody. c Quantification of secreted HA-TDP-43-K263E in conditioned media. Data represent mean ± SEM, *n* = 6 experiments. Significance was assessed by a Mann-Whitney U test (**p = 0.0022).

**Supplementary Fig. S7** TDP-43-K263E partially colocalizes with USP19 and LC3**. a-d** HEK293T cells co-expressing HA-TDP-43-K263E and Flag-USP19-WT were analyzed 36 h after transfection by confocal immunofluorescence using anti-HA (red/HA-TDP-43), anti-LC3 (green/LC3), anti-Flag (purple/Flag-USP19) and DAPI for nuclear staining (blue). Scale bar is 5μm.

**Supplementary Fig. S8** The TDP-43 is embedded into cytoplasmic compact electron dense structures (CEDS) in cells overexpressing the Flag-USP19-ΔTM. TDP-43 IEM of HEK293T TDP-43-K263E and Flag-USP19-ΔTM co-expressing cells. Left panel: the black arrow indicates the cytoplasmic CEDS surrounded by the dotted line. Red arrows indicate the TDP-43 gold particle labelling embedded into the CEDS. Right panel: note that TDP-43 gold particle labelling (red arrows) is slightly higher into the CEDS.

**Supplementary Fig. S9** Kinetic of TDP-43-K263E secretion upon Flag-USP19-WT overexpression. Kinetic of TDP-43-K263E secretion in Flag-USP19-WT (left panel) or Flag-USP19-ΔTM (right panel) contexts. Conditioned media from co-expressing cells were recovered at 24, 27, 30, 33, 36 and 40 h after transfection and submitted to FTA. Nitrocellulose membranes were probed with anti-TDP-43. Western blotting analyses of cell lysates from co-expressing cells using antibodies directed against the Flag (for Flag-USP19s), HA (HA-TDP-43-K263E), LC3 (LC3I and II), and GAPDH for loading control.

**Supplementary Fig. S10** Vps34 class III PI3K inhibitors significantly reduce misfolded TDP-43 secretion upon Flag-USP19-WT overexpression. **a** Evaluation of the TDP-43-K263E secretion mediated by USP19 by FTA in presence or absence of Bafilomycin A1 (Baf A1, 5 nM), Chloroquine (CQ, 50 mM), Spautin-1 (1 mM), LY294002 (50 µM) pharmacological inhibitors or DMSO as negative control. Upper panel (secretion): nitrocellulose membranes were probed with an antibody directed against TDP-43. Lower panel (cell expression): Western blotting of cell lysates from drugs-treated co-expressing cells using antibodies directed against Flag (for USP19), TDP-43, LC3 (LC3 I and II), S6 and phospho-S6 and GAPDH for loading control. **b** Quantification of secreted K263E-TDP-43 in presence or absence of compounds. Data represent mean ± SEM, *n* = 5 to 12 experiments. Significance was assessed by a Mann-Whitney U test (**p < 0.001; ***p<0.0001).

**Supplementary Fig. S11** No synergistic effect of VAMP7-WT and USP19-WT on misfolded GFP-TDP-43-ΔNLSΔ187-192 secretion. **a** Evaluation of misfolded TDP-43 secretion by FTA upon combined expression of Flag-USP19-WT and RFP-VAMP7-WT. **b** representative FTA showing all the experimental conditions on the same nitrocellulose membrane. **c** Data represent mean ± SEM, n=3. Significance was assessed by a Mann-Whitney U test. ns non-significant.
